# Peptidomics Analysis Reveals Peptide PDCryab1 Inhibits Doxorubicin-Induced Cardiotoxicity

**DOI:** 10.1155/2020/7182428

**Published:** 2020-10-13

**Authors:** Li Zhang, Xuejun Wang, Mengwen Feng, Hao Zhang, Jia Xu, Jingjing Ding, Zijie Cheng, Lingmei Qian

**Affiliations:** ^1^Department of Cardiology, The First Affiliated Hospital of Nanjing Medical University, Nanjing 210029, China; ^2^Department of General Practice, Tongren Hospital, Shanghai Jiao Tong University School of Medicine, 1111 Xianxia Road, Shanghai 200336, China; ^3^Department of Cardiology, Tongren Hospital, Shanghai Jiao Tong University School of Medicine, 1111 Xianxia Road, Shanghai 200336, China

## Abstract

Doxorubicin (DOX) is limited due to dose-dependent cardiotoxicity. Peptidomics is an emerging field of proteomics that has attracted much attention because it can be used to study the composition and content of endogenous peptides in various organisms. Endogenous peptides participate in various biological processes and are important sources of candidates for drug development. To explore peptide changes related to DOX-induced cardiotoxicity and to find peptides with cardioprotective function, we compared the expression profiles of peptides in the hearts of DOX-treated and control mice by mass spectrometry. The results showed that 236 differential peptides were identified upon DOX treatment, of which 22 were upregulated and 214 were downregulated. Next, we predicted that 31 peptides may have cardioprotective function by conducting bioinformatics analysis on the domains of each precursor protein, the predicted score of peptide biological activity, and the correlation of each peptide with cardiac events. Finally, we verified that a peptide (SPFYLRPPSF) from Cryab can inhibit cardiomyocyte apoptosis, reduce the production of reactive oxygen species, improve cardiac function, and ameliorate myocardial fibrosis in vitro and vivo. In conclusion, our results showed that the expression profiles of peptides in cardiac tissue change significantly upon DOX treatment and that these differentially expressed peptides have potential cardioprotective functions. Our study suggests a new direction for the treatment of DOX-induced cardiotoxicity.

## 1. Introduction

Doxorubicin (DOX), a typical broad-spectrum and highly effective antitumor drug, is widely used in the clinical treatment of various malignant tumors, such as breast cancer, lymphoma, and leukemia [1]. However, its widespread clinical application is limited by cumulative dose-dependent toxicity in multiple organs, especially cardiotoxicity [2]. Research has shown that DOX can induce chronic heart failure when the cumulative clinical dose exceeds 400-700 mg/m^2^ (adult) or 300 mg/m^2^ (child), which greatly limits the dose of DOX for clinical treatment [3]. It is currently recognized that the main mechanism of DOX-induced cardiotoxicity is oxidative stress and the apoptosis of cardiomyocytes [4, 5]. DOX can induce cardiomyocyte apoptosis, which can develop into chronic heart failure, through the generation of a large amount of reactive oxygen species and cell calcium overload because of its high affinity for myocardial tissue and tendency for accumulation in cardiomyocytes [6, 7]. Currently, no drugs except dexrazoxane can be utilized clinically to prevent or cure DOX-induced cardiotoxicity. Therefore, to find an intervention strategy, it is necessary to explore the mechanism of cardiotoxicity caused by DOX from a new perspective.

Peptidomics, an emerging field of proteomics [8], is a method for comprehensively analyzing peptides in various biological samples by mass spectrometry [9, 10]. It can be used for systematically, qualitatively, and quantitatively studying the composition and content of endogenous peptides in organisms under physiological or pathological conditions. With the development of peptidomics, a class of small-molecule peptides composed of 3-50 amino acids has been found to be important participants in a variety of life activities, including apoptosis [11], immune regulation [12], cell differentiation [13], nervous system regulation [14], and reproduction regulation [15], and because of their advantages, such as easy synthesis, small molecular weight, nontoxic metabolites, and easy access to cells, they have become a new favorite in the field of drug research and development [16]. Humanin, a 24 amino acid peptide, is encoded by the open reading frame in the mitochondrial 16S rRNA region and has shown cardiomyocyte protection and antioxidant and antiapoptosis properties [17, 18]. Humanin can enhance the cardioprotective effect of dexrazoxane on DOX-induced cardiotoxicity, which may indicate its use as an adjuvant for dexrazoxane to reduce DOX-induced cardiotoxicity [19]. Exenatide pretreatment inhibits DOX-induced production of reactive oxygen species and apoptosis in cardiomyocytes and improves cardiac dysfunction through the upregulation of autophagy, indicating its therapeutic potential for preventing DOX-induced cardiotoxicity [20]. Apelin is an endogenous peptide ligand of the APJ receptor that can prevent the activation of cardiac fibroblasts and the production of collagen by inhibiting sphingosine kinase 1. In addition, the use of apelin in the stage of reactive fibrosis can prevent myocardial structural remodeling and ventricular dysfunction [21]. Therefore, considering peptides, we may find new clues for the protection of DOX-induced cardiotoxicity.

In this study, we established a cardiotoxicity model by continuous doxorubicin injection. Nano-LC-MS/MS mass spectrometry was utilized to explore the dynamic changes in the composition of the endogenous peptides in mouse heart tissue and screen for potentially functional peptides related to DOX-induced cardiotoxicity. Subsequently, we analyzed the differentially expressed peptides using a bioinformatics approach and predicted 31 peptides that may have cardioprotective functions. Finally, a peptide derived from Cryab was verified to antagonize cardiomyocyte apoptosis and reduce ROS production. This study used peptidomics as an entry point to explore the means of preventing or ameliorating the cardiotoxicity caused by doxorubicin, providing new ideas for the study of DOX-induced cardiotoxicity.

## 2. Materials and Methods

### 2.1. Mice

Six-week-old male C57BL/6 mice weighing 16-20 g were purchased from the Shanghai Slake Experimental Animal Co., Ltd., and raised at the SPF Laboratory Animal Center of Nanjing Medical University. The experiment was started one week after the animals were purchased. The experimental animals were randomly allocated to two groups: the control group and the DOX group, with 12 mice in each group. In the DOX group, the mice were injected intraperitoneally with 5 mg/kg DOX a week for a total of 4 injections with a cumulative dose of 20 mg/kg [22], while the control group was injected intraperitoneally with an equal volume of saline. All animal experiments were carried out in accordance with the Guide for the Care and Use of Laboratory Animals published by the National Institutes of Health (NIH Publications No. 85-23, revised 1996) and reviewed by the Animal Experiment Ethics Committee of Nanjing Medical University (Nanjing, China).

### 2.2. Echocardiography and Histological Determination

After DOX or saline administration, the animals were maintained for 2 weeks; mice were lightly anesthetized with 1.5% isoflurane and allowed to breathe spontaneously, and then echocardiography was performed to detect mouse cardiac function. High-resolution small animal ultrasound imaging system (Vevo 3100) was used to obtain M-mode ultrasound measurements for the DOX injection group and normal group mice. The main measurement indicators included ejection fraction (EF) and fractional shortening (FS). Other echocardiographic parameters including left ventricular end-systolic diameter (LVEDs), left ventricular end-diastolic diameter (LVEDd), left ventricular end-systolic anterior wall thickness (LVAWs), and left ventricular end-diastolic anterior wall thickness (LVAWd). Further, left ventricular fractional shortening (FS%) was calculated as [(LVEDd − LVEDs)/LVEDd] × 100; left ventricular ejection fraction (EF%) was calculated as [(LVEDV − LVESV)/LVEDV] × 100, LVEDV = [7 LVEDd^3^/(2.4 + LVEDd)], and LVESV = [7 LVEDs^3^/(2.4 + LVEDs)]. After the mice were euthanized by carbon dioxide asphyxiation, the ventricular tissue was collected and immediately fixed in 4% paraformaldehyde for 48 hours. Samples were dehydrated, paraffin embedded, and sectioned into 5 *μ*m thick slices on a sliding microtome (Leica, Nussloch, Germany). Then, the myocardial sections were dewaxed, rehydrated, and stained with Masson's trichrome, and the degree of myocardial fibrosis was observed under the microscope. Blue collagen staining was quantified using ImageJ software (version 1.52t, National Institutes of Health, Bethesda, MD, USA).

### 2.3. Peptide Extraction

The heart tissue samples were added with Tris-HCl according to the volume ratio of 1 : 3, heated and boiled for 10 min, then cooled in an ice water bath, and then broken by ultrasonic wave at 100 Hz for more than 5 s, with an interval of 5 s and ultrasonication for 2 min. Then, the final concentration of 1 M glacial acetic acid was added into the sample tube, and vortex oscillation was performed for 2 min. Then, acetonitrile with final concentration of about 50% was added. The sample tube was centrifuged at 12000 × g at 4°C for 10 min; after that, the supernatant was transferred to a clean EP tube for freeze-drying. Next, add 80% acetone solution, vortex, vibrate, ultrasonicate in a water bath for 2 min and 4°C, centrifugate at high speed at20000 × gfor 30 min, and then take the supernatant and transfer to a clean EP tube for freeze-drying. Finally, add 200 *μ*l of 0.1% TFA solution for redissolution and remove salt with C18 with sample loading of 80 *μ*g, freeze-dry, and set aside.

### 2.4. LC/MS and Peptide Identification

We considered that the heart of mice was small. In order to make the sample quality detected by mass spectrometry more sufficient, we used the hearts of four mice to mix into one sample. Four mouse heart tissues of DOX treatment were mixed as one DOX sample and four normal mouse heart tissues were mixed as one control sample, a total of three groups of DOX and three groups of control. The peptides were identified by nano-LC-MS/MS on a Q Exactive Plus mass spectrometer (Thermo) couple with LC1000. Solvent A (Milli-Q [Millipore, Billerica, MA] water with 0.1% formic acid and 2% acetonitrile) and solvent B (90% acetonitrile with 0.1% formic acid) were used for chromatographic separation. The peptides were eluted with 5% solvent B for 5 minutes at a rate of 300 nl/min, 5-40% solvent B for 65 minutes, 40-80% solvent B for 1 minute, 80% solvent B for 4 minutes, and then 5% solvent B over 20 minutes. Q Exactive Plus (Thermo Fisher) was performed in an information-dependent data acquisition mode to enable automatically switching between MS and MS/MS acquisition. MS spectra were obtained in the mass range of 350-2000 m/z. Xcalibur software (Thermo Scientific, version 3.1.66.10) was used for automatic peak identification, 10 s dynamic exclusion, and tandem mass spectrometry analysis of the top 20 precursor protein ions at 30% normalized collision energy. The intensities of identified peptides were calculated by MaxQuant software (version 1.6.6.0) and used label-free quantification.

All MS/MS data were analyzed by MaxQuant software, and the UniProt_mouse database (UniProt release 2019_11) was searched based on nonspecific digestion technology. The mass tolerance of the fragment ion in MaxQuant was 0.050 Da and that of the parent ion was 10.0 PPM. Oxidation of methionine was designated as a variable modification. The selection criterion for the differentially expressed peptides was a fold change larger than 2 with a *P* value < 0.05 (Student's *t*-test).

### 2.5. Bioinformatics Analysis

The peptide isoelectric point (PI) and molecular weight (MW) information were obtained online (https://web.expasy.org/protparam/). First, the UniProt database (http://www.uniport.org/) was used to find the source of differential peptides. Functional Annotation Tool DAVID Bioinformatics Resources 6.8 (https://david.ncifcrf.gov/) was used to elucidate the potential functions of the precursor proteins of the identified peptides according to the biological process, molecular function, and cellular component categories of Gene Ontology (GO) annotations and KEGG pathways. Second, the relationship of the differential peptides' precursor proteins with various cardiovascular diseases and apoptosis was analyzed using the GeneAnalytics website (http://geneanalytics.genecards.org/) and Cytoscape 3.5.1 software. Third, the biological activity of peptides was predicted through Peptide Ranker [23, 24] (http://bioware.ucd.ie/~compass/biowareweb/, ranker scores greater than 0.5 indicate possible activity) in order to find potential peptides. The protein interactions were analyzed using the STRING website (https://string-db.org/, version: 11.0) and Cytoscape 3.5.1 software. The amino acid sequences of the different species were analyzed using the protein database on the NCBI website (https://www.ncbi.nlm.nih.gov/homologene/), and the results were compared with DNAMAN (version 9.0) software.

### 2.6. Peptide Synthesis and Administration

The following peptide sequences were synthesized in this experiment: PDCryab1: RKKRRQRRR-SPFYLRPPSF, PDCryab2: RKKRRQRRR-SPFYLRPPSFLR, PDCryab3: RKKRRQRRR-SPFYLRPPSFLRAPS, PDCryab4: RKKRRQRRR-TSLSPFYLRPPSFL, and scramble peptide of PDCryab1: RKKRRQRRR-LSFRFPSPYP. We used scramble peptide to serve as the control peptide, which shares same amino acids with Cryab but sequenced as a scramble peptide.

The RKKRRQRRR sequence is a cell-penetrating peptide composed of nine amino acids in HIV-1 Tat (49-57). The purity of all peptides was more than 95%. The peptides were synthesized by the Shanghai Science Peptide Biological Technology Co., Ltd. (Shanghai, China). The peptide crystal was dissolved in sterile water to obtain a storage solution of 10 mM and diluted to the final concentration corresponding to the experiment. For the cell experiments, peptides were added to the culture supernatant 2 hours before DOX treatment.

### 2.7. Cell Culture and Experimental Design

The H9c2 cell line of rat cardiomyocytes was purchased from the Cell Bank of the Shanghai Academy of Biosciences. H9c2 cells were cultured in a sterile incubator (37°C, 5% CO_2_) with high-glucose DMEM supplemented with 10% fetal bovine serum and 1% penicillin and streptomycin. The H9c2 cells were subcultured every 2 days and were in good condition for use in the experiments.

For the initial cell experiments, the cells were allocated to 6 groups: (I) control group, (II) DOX (1 *μ*M) treatment group (DOX), (III) DOX and PDCryab1 (50 *μ*M) [25, 26] cotreatment group (DOX+P1), (IV) DOX and PDCryab2 (50 *μ*M) cotreatment group (DOX+P2), (V) DOX and PDCryab3 (50 *μ*M) cotreatment group (DOX+P3), and (VI) DOX and PDCryab4 (50 *μ*M) cotreatment group (DOX+P4). In addition, for the subsequent cell experiments, the cells were allocated to the following groups: (I) control group, (II) PDCryab1 (50 *μ*M) treatment group, (III) DOX (1 *μ*M) treatment group (DOX), (IV) DOX and PDCryab1 (10 *μ*M) cotreatment group (10), (V) DOX and PDCryab1 (20 *μ*M) cotreatment group (20), and (VI) DOX and PDCryab1 (50 *μ*M) cotreatment group (50). After 24 hours of these treatments, the cells were collected for the appropriate analysis.

### 2.8. Analysis of Cell Viability

Cell viability was determined with a cell counting kit-8 (CCK-8) kit (Dojindo Molecular Technologies, Inc., Kumamoto, Japan) according to the following steps. The subcultured H9c2 cells were seeded in 96-well plates at a density of 1 × 10^4^ cells/well, and upon reaching the adherence stage, the cells were treated with drugs as described above. Ten microliters of the CCK-8 reagent was added to each well and maintained away from light in 37°C incubators. After incubation for 2 hours, the intensity of the light absorption at 450 nm wavelength was measured by a microplate reader.

### 2.9. Western Blot Analysis

The protein in the H9c2 cells was extracted with lysis buffer (including RIPA and 1% PMSF) and quantified by a BCA assay kit (23229; Thermo Fisher Scientific). The protein samples were mixed with 1x SDS loading buffer and denatured by boiling at 95°C for 5 minutes. After cooling on ice for 4 minutes, the protein samples were separated by 10% SDS-PAGE gel and then transferred to nitrocellulose membranes (Millipore, USA). After being blocked with 5% skimmed milk at room temperature for 1.5 hours, specific antibodies, namely, anti-PARP (1 : 1000, #9542, CST), anti-caspase3 (1 : 1000, #9665, CST), *β*-actin (1 : 2000, #4970, CST), and antitubulin *α*/*β* (1 : 2000, #2148, CST), were incubated with the membrane overnight at 4°C. The membrane was washed once with TBST buffer 5 times for 5 minutes each time. Protein expression was quantitatively analyzed by Image Lab software (Bio-Rad, Hercules, CA, USA). Western blot results were normalized by tubulin or *β*-actin.

### 2.10. Detection of ROS, SOD, and MDA

Reactive oxygen species (ROS) were detected by an ROS assay kit (Beyotime, China) according to standard procedures. H9c2 cells were passaged to a 6-well plate at a density of 2 × 10^5^ cells/well. After the cells reached 80% confluence, the cells were treated with drugs for a specified time. The DCFH-DA fluorescence probe was diluted to 10 *μ*M in DMEM without serum. The 6-well plate medium was transferred to a clean centrifuge tube for preservation, and 1 ml of diluted DCFH-DA fluorescence probe was added to each well and incubated at 37 °C in the dark for 20 minutes. After incubation, the supernatant was discarded, and the cells were washed twice with serum-free DMEM medium and then washed twice with PBS. The ROS fluorescence intensity in the cells was observed by inverted fluorescence microscopy and quantified by ImageJ software. SOD and MDA assay kits (Nanjing Jiancheng Biocompany) are used to detect SOD and MDA levels in the cell supernatant that has been retained according to the protocol.

### 2.11. Mitochondrial Membrane Potential

JC-1 Mitochondrial Membrane Potential Assay Kit (Beyotime, China) was used to analyze mitochondrial injury according to the manufacturer's instructions. In short, the cells were washed with PBS and incubated with JC solution for 10 min at 37°C. And then, the cells were washed with dilution buffer again and analyzed on a laser scanning confocal microscope.

### 2.12. TUNEL Assay

Cells were seeded (1 × 10^5^ cells per well) in 6-well plates. After DOX treatment, the cells were washed twice with PBS and fixed with 4% paraformaldehyde. Apoptotic cells were visualized with TUNEL staining according to the manufacturer's protocol (Promega). TUNEL fluorescence intensity/DAPI fluorescence density was used to calculate the percentage of positive cells, and the density was evaluated using ImageJ software 1.26 (Wayne Rasband, National Institutes of Health, Bethesda, MD, USA).

### 2.13. LDH Release

The level of LDH was detected by LDH Release Assay Kit (Beyotime, China). The reaction solution was prepared according to the manufacturer's instructions. The cell supernatant or serum (120 *μ*l/well) was collected and mixed with reaction solution (60 *μ*l/well), and then the mixtures were added into 96-well plates. The cells were wrapped in tin foil and incubated for 30 min at RT on the shaker. Finally, the absorbance was detected with a microplate reader at 490 nm wavelength.

### 2.14. Histological Staining

Hearts were harvested and immediately fixed in 4% paraformaldehyde for 48 hours. Samples were dehydrated, paraffin embedded, sectioned into 5 *μ*m thick slices on a sliding microtome (Leica, Nussloch, Germany), and stained with Sirius red and hematoxylin and eosin (H&E). The yocyte cross-sectional areas were measured via fluorescein isothiocyanate-conjugated WGA (L4895; Sigma, St. Louis, MO, USA) staining. A quantitative digital image analysis system (Image-Pro Plus 6.0) was used to measure the cross-sectional area of the cardiomyocyte from images that had been captured from fluorescein isothiocyanate- (FITC-) conjugated wheat germ agglutinin- (WGA-) (Invitrogen, Thermo Fisher Scientific) stained sections.

### 2.15. Real-Time PCR

Total RNA was extracted from cells using TRIzol reagent (Thermo Fisher Scientific). The concentration of RNA was determined by measuring the absorbance ratio of 260/280 nm using a NanoDrop ND-1000 spectrophotometer (Thermo Scientific). Reverse transcription of RNA was performed using a PrimeScript™ RT Reagent Kit with gDNA eraser (RR047A; Takara, Tokyo, Japan), and cDNA was analyzed by qRT-PCR using SYBR® Premix Ex Taq™ (RR420A; Takara, Tokyo, Japan). The data were normalized to the levels of GAPDH and further analyzed using the 2^−*ΔΔ*CT^ method.

### 2.16. Statistical Analysis

The experimental data and statistical graphs were analyzed by GraphPad Prism 8 software. All data are presented as the means ± standard deviation (SD). Statistical differences were measured with an unpaired 2-sided Student *t*-test or 2-way ANOVA with Bonferroni correction for multiple comparisons. When the *P* value < 0.05, the difference was considered significant.

## 3. Results

### 3.1. Peptidomics Research Process Using Mouse Heart Tissue

**Figure 1:**
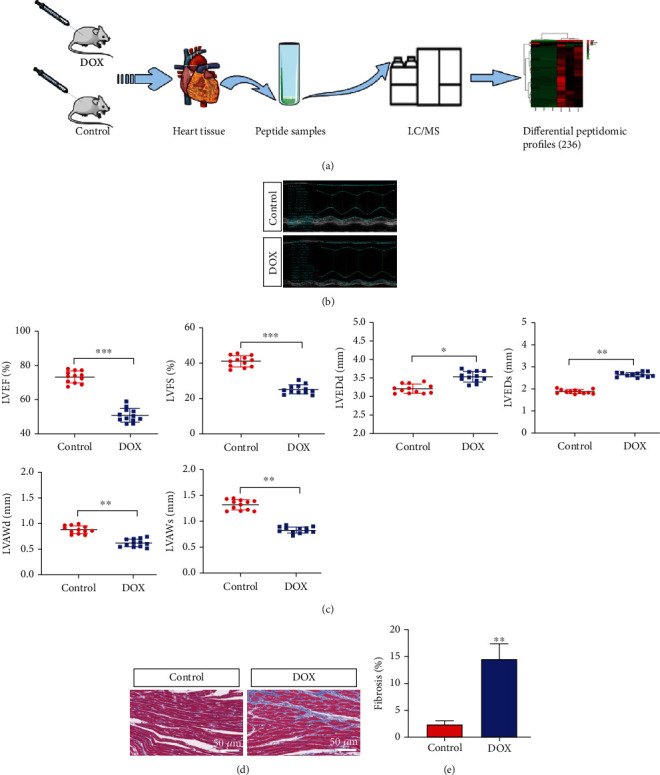
Peptidomics research process and the successful construction of a mouse model. (a) The process of peptide identification in the mouse heart tissues by LC/MS mass spectrometry. (b) Representative M-mode echocardiography-derived graphs of the DOX group and control group. (c) Quantitative data echocardiography analysis (*N* = 12 mice per group). (d, e) Representative photographs of Masson's trichrome staining show cardiac fiber rupture and decreased cardiomyocyte area in the DOX group. Quantification data of Masson's trichrome staining. Magnified 400x. The data represent the means ± SD. ^∗∗∗^*P* < 0.001.

We used male C57BL/6 mice to construct an animal model of cardiotoxicity by intraperitoneally injecting DOX and then collecting heart tissues to extract peptides for mass spectrometry analysis. The schematic process is shown in Figure 1(a). Dox-induced cardiac dysfunction was remarkably decreased in the Dox injection group, which was indicated by a decrease in EF and FS (Figures 1(b) and 1(c)). Masson's staining results showed the cardiac fibrosis alterations, as evidenced by cardiac fiber rupture and decreased cardiomyocyte area (Figures 1(d) and 1(e)). Thus, we established a cardiotoxicity model induced by DOX injection.

### 3.2. Identification of Differential Peptide Expression Profiles Related to DOX-Induced Cardiotoxicity

**Figure 2:**
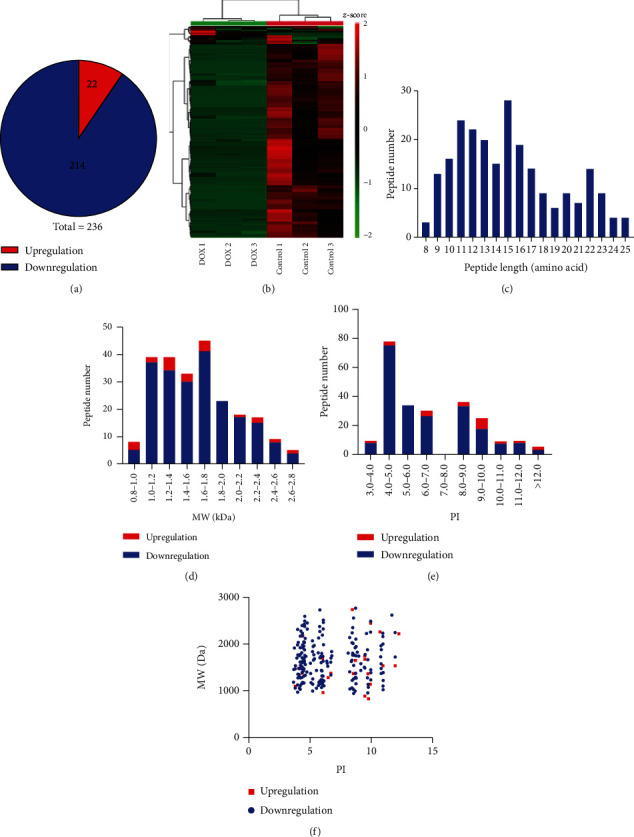
Identification and features of differentially expressed peptides. (a) Identification of the number of differentially expressed peptides. (b) Heat map of the differentially expressed peptides. Red indicates upregulation, and green indicates downregulation. (c) Distribution of the differentially expressed peptides by length. (d) Molecular weight (MW) of the differentially expressed peptides. (e) Isoelectric point (PI) of the differentially expressed peptides. (f) The correlation between the distribution of differentially expressed peptides by MW and PI.

Mass spectrometry results revealed 2945 detected peptides, 236 of which were differentially expressed (*P* value < 0.05 and fold change ≥2) (Supple. Table 1). In the DOX-induced cardiotoxicity group, 22 peptides were upregulated and 214 peptides were downregulated (Figure 2(a)). The heat map shows the significant differences in the peptide profiles of cardiotoxic tissues treated with doxorubicin and normal heart tissues (Figure 2(b)). 47 peptides were expressed exclusively in the control group and 5 peptides were expressed in the DOX group (Supple. Table 2). Three peptides possessing 7 different precursor proteins are listed in Supple. Table 3. We found that the distribution of different peptide lengths was relatively large and mainly concentrated in two ranges: 9-17 and 22-23 amino acids (Figure 2(c)). We also explored the molecular weight (MW) and isoelectric point (PI) of differentially expressed peptides and found that the MW of the differentially expressed peptides was distributed between 0.8 and 2.8 kDa, with the downregulated peptides mainly concentrated in the 1.0-2.0 kDa range and the upregulated peptides concentrated in the 0.8-1.0 kDa and 1.2-1.8 kDa ranges (Figure 2(d)). The isoelectric point analysis showed that, overall, the differentially expressed peptides were mainly in the PI 4-7 and PI 8-10 ranges (Figure 2(e)). The distribution of upregulated and downregulated peptides was consistent with that of all peptides, and there was no significant difference in the distribution between the upregulated and downregulated groups. In addition, we analyzed the correlation between the distribution of differential peptide MW and PIs. The peptides were mainly clustered into four groups: near PI 4, PI 6, PI 8, and PI 10 (Figure 2(f)).

### 3.3. Analysis of Four Cleavage Sites in the Differentially Expressed Peptides

**Figure 3:**
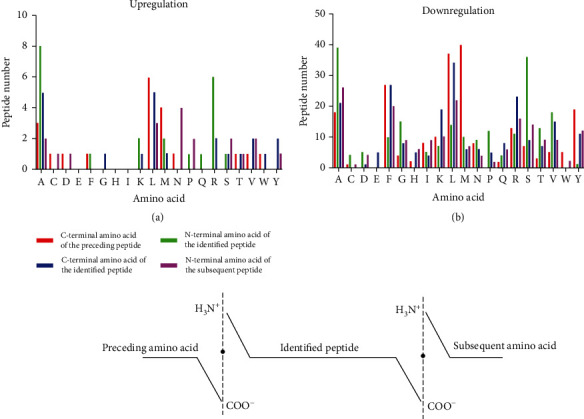
Analysis of four cleavage sites of the differentially expressed peptides. (a) The distribution of the four cleavage sites in the upregulated peptides. (b) The distribution of four cleavage sites in the downregulated peptides.

Based on the peptide described data, we analyzed the C-terminal and N-terminal cleavage sites of the differentially expressed peptides, which mainly included the following four cleavage sites: the C-terminal amino acid of the preceding peptide, the N-terminal amino acid of the identified peptide, the C-terminal amino acid of the identified peptide, and the N-terminal amino acid of the subsequent peptide. In the upregulated peptide group, leucine (L) was the most abundant amino acid at the C-terminus of the preceding peptide, alanine (A) was the most abundant amino acid at the N-terminus of the identified peptide, alanine (A) and leucine (L) were the most abundant amino acids at the C-terminus of the identified peptide, and asparagine (N) was the most abundant amino acid at the N-terminus of the subsequent peptide (Figure 3(a)). In the downregulated group, the most abundant amino acids in the above four cleavage sites were methionine (M), alanine (A), leucine (L), and alanine (A), as shown in Figure 3(b). The four cleavage sites of 236 peptides were different in the upregulated and downregulated groups.

### 3.4. Bioinformatics Analysis

**Figure 4:**
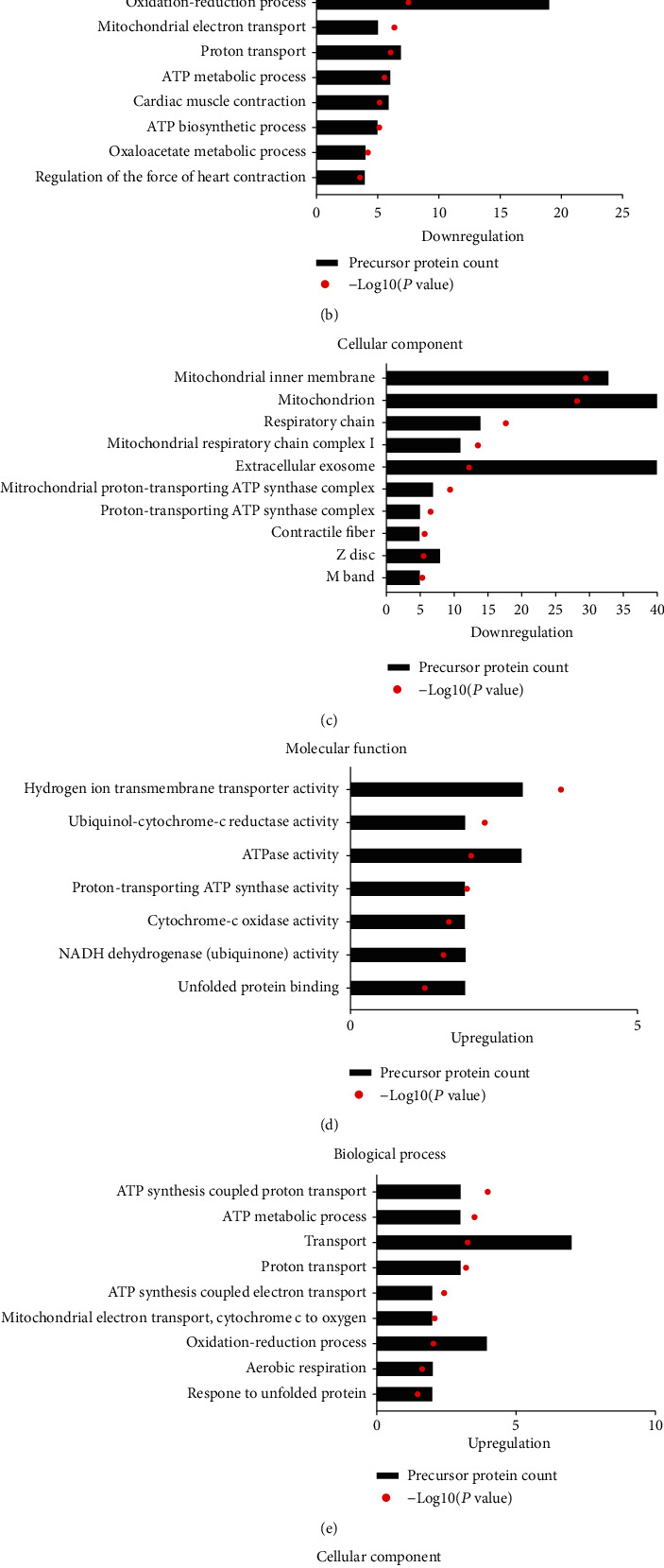
Gene Ontology (GO) and pathway analysis. (a) Molecular function of downregulated peptides' precursor proteins. (b) Biological processes of downregulated peptides' precursor proteins. (c) Cellular components of downregulated peptides' precursor proteins. (d) Molecular function of upregulated peptides' precursor proteins. (e) Biological processes of upregulated peptides' precursor proteins. (f) Cellular components of upregulated peptides' precursor proteins.

**Figure 5:**
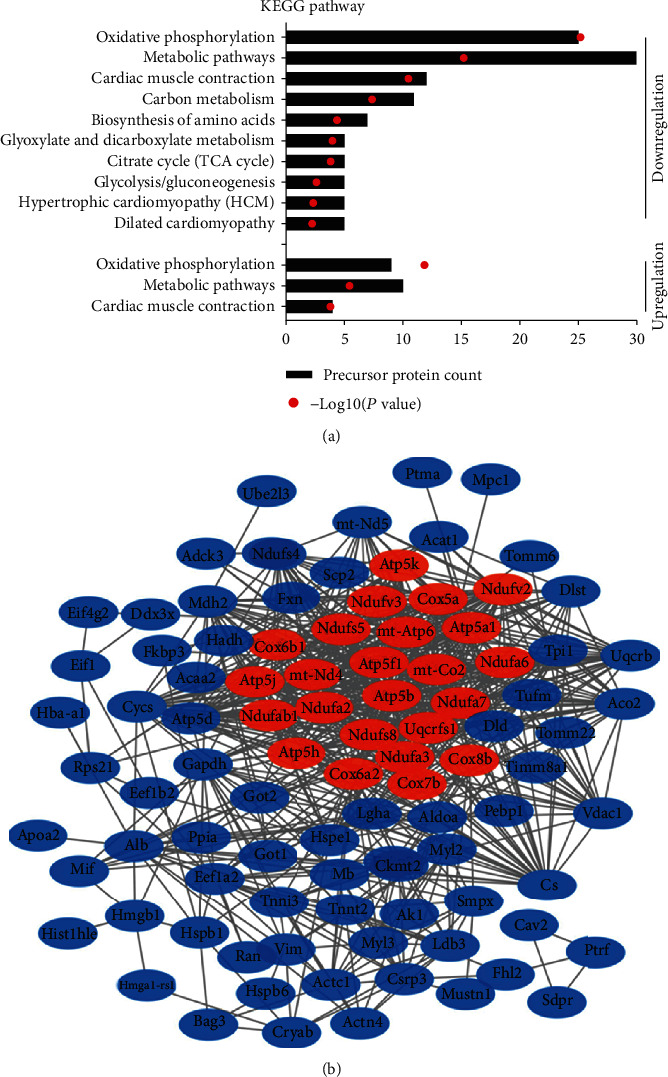
Analysis of the protein interaction network. (a) Pathway analysis of the precursor proteins of the differentially expressed peptides. (b) Interaction network of precursor proteins of these differentially expressed peptides as determined with STRING (https://string-db.org/, version: 11.0). The confidence level: medium confidence 0.400. Red: the main proteins associated with oxidative phosphorylation.

To predict the potential function of 236 differentially expressed peptides, we performed GO and pathway analysis on their precursor proteins. GO analysis results showed the molecular function, biological process, and cellular component in the downregulation proteins (Figures 4(a)–4(c)). Interestingly, we found that downregulated proteins were mainly associated with Poly(A) RNA binding, transport, and mitochondrial function. Downregulated protein analysis showed transmembrane transporter activity, ATP synthesis, mitochondrial respiratory chain (Figures 4(d)–4(f)). The KEGG pathway analysis showed that the precursor proteins of the differential peptides were mainly involved in oxidative phosphorylation and metabolism signaling pathways, which are closely related to the occurrence and development of myocardial injury (Figure 5(a)). Next, we analyzed the interaction network of the precursor proteins of these differential peptides using the STRING website (https://string-db.org/, version: 11.0). We found multiple interaction networks, with the main protein interaction network related to oxidative phosphorylation in mitochondria (Figure 5(b)).

### 3.5. Prediction of Myocardial Protective Peptides

**Figure 6:**
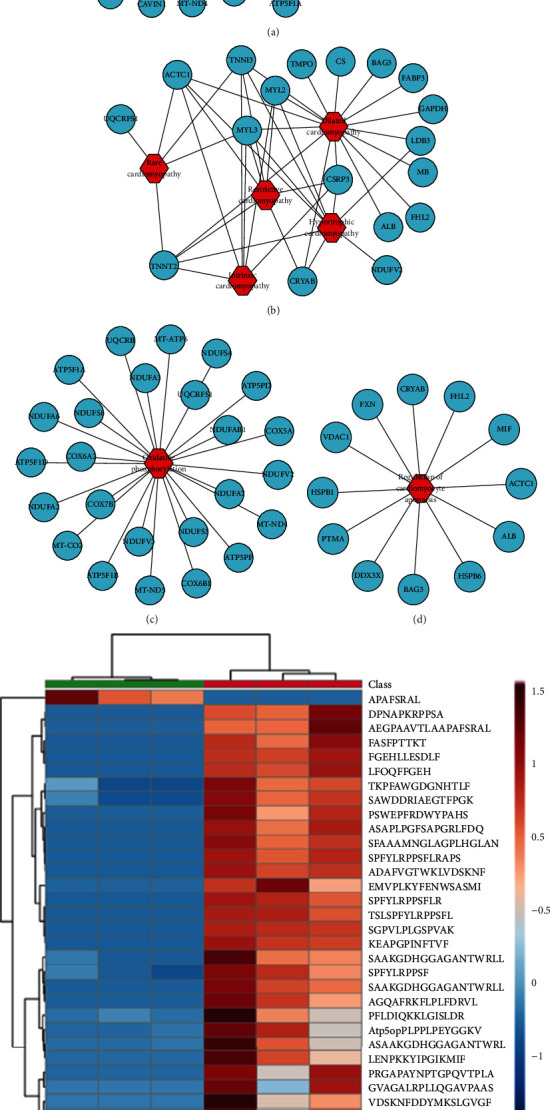
Analysis of the precursor proteins of differentially expressed peptides related to potential myocardial protection. (a) Analysis of these precursor proteins related to cardiovascular diseases. (b) Analysis of these precursor proteins related to various cardiomyopathies. (c, d) Analysis of these precursor proteins related to oxidative phosphorylation and cardiomyocyte apoptosis. (e) Heat map analysis of 31 peptides that may confer myocardial protection.

First, we sought to determine the precursor proteins of the differentially expressed peptides related to cardiovascular diseases, oxidative phosphorylation, and cardiomyocyte apoptosis through the GeneAnalytics website (http://geneanalytics.genecards.org/) and Cytoscape 3.5.1 software. The correlations between the precursor proteins and various cardiovascular diseases are shown in Figure 6(a). The correlation between the precursor proteins and cardiomyopathy is shown in Figure 6(b). The precursor proteins associated with oxidative phosphorylation and cardiomyocyte apoptosis are shown in Figures 6(c) and 6(d). Next, we used the UniProt database (https://www.UniProt.org/) to study the function of the differentially expressed peptides and their precursor proteins and used Peptide Ranker (http://bioware.ucd.ie/~compass/biowareweb/, ranker scores greater than 0.5 indicate possible activity) to predict the probability that a differentially expressed peptide is involved in a biological activity. Finally, we screened 31 differentially expressed peptides that may have myocardial protective function (Table 1). The heat map shows small differences within the group of 31 peptides and large differences between the behavior activity groups (Figure 6(e)).

### 3.6. Preliminary Functional Exploration of Peptides Derived from Cryab

**Figure 7:**
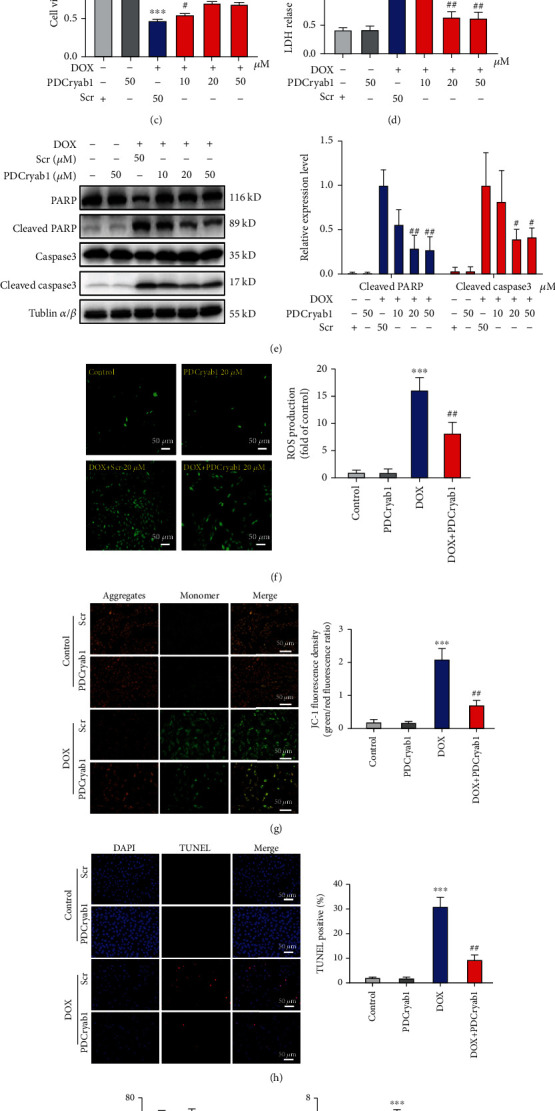
PDCryab1 attenuates DOX-induced cardiomyocyte apoptosis and the generation of reactive oxygen species (ROS). (a) The protein level of Cryab was significantly decreased after DOX treatment. (b) The effect of four peptides derived from Cryab on the viability of cells after DOX treatment, as determined by CCK-8 assay (one-way ANOVA analysis with Bonferroni's multiple comparison test). (c) The effect of PDCryab1 at different concentrations on the viability of cells after DOX treatment, as determined by CCK-8 assay (two-way ANOVA analysis with Bonferroni's multiple comparison test). (d) Treatment of PDCryab1 significantly reduced LDH release (two-way ANOVA analysis with Bonferroni's multiple comparison test). (e) Western blot analysis of cleaved PARP and cleaved caspase3. The quantification data for cleaved PARP and cleaved caspase3 (two-way ANOVA analysis with Bonferroni's multiple comparison test). (f) Representative photographs of ROS stained in the H9c2 cells. A DCFH-DA probe was used to detect intracellular ROS. Magnification 100x. The peptide concentration was 20 *μ*M. Green: ROS. Quantification data for the ROS (two-way ANOVA analysis with Bonferroni's multiple comparison test). (g) Representative photographs of mitochondrial membrane potential in H9c2 cells (two-way ANOVA analysis with Bonferroni's multiple comparison test). Magnification 200x. (h) Representative photographs of TUNEL in H9c2 cells (two-way ANOVA analysis with Bonferroni's multiple comparison test). Magnification 100x. (i) SOD and MDA were detected (two-way ANOVA analysis with Bonferroni's multiple comparison test). The data represent the means ± SD. ^∗∗^*P* < 0.01 and ^∗∗∗^*P* < 0.001 versus the control group. ^#^*P* < 0.05 and ^##^*P* < 0.01 versus the DOX/DOX+Scr group. ns: not statistically significant.

Research has shown that Cryab protein plays an important role in myocardial protection. First of all, we verified the protein level of Cryab before and after DOX treatment. Our results showed that the protein level of Cryab was significantly reduced after DOX treatment (Figure 7(a)). Thus, we wondered if it might have a cardioprotective effect by cracking down key peptides. Among the 31 predicted peptides, six peptides were derived from Cryab and downregulated in the DOX group. Therefore, we selected four peptides that have a relatively high ranker score for the preliminary functional experiments with H9c2 cells: SPFYLRPPSF (45-54), SPFYLRPPSFLR (45-56), SPFYLRPPSFLRAPS (45-59), and TSLSPFYLRPPSFL (42-55). We named these peptides PDCryab1-4 (peptides derived from Cryab). Subsequently, we preliminarily verified the functions of these four peptides in H9c2 cells. We confirmed the PDCryab1 (SPFYLRPPSF) peptide to significantly enhance the viability of DOX-treated cardiomyocytes (Figure 7(b)). The analysis of PDCryab1 (SPFYLRPPSF) conservation in various species is shown in Supple. Fig. 1A. PDCryab1 has a high degree of homology among various species, especially *Homo sapiens*, mouse, and Rattus. HCD MS/MS annotation of the PDCryab1 peptide (SPFYLRPPSF) derived from the Cryab sequence spanning amino acids 45-54 is shown in Supple. Fig. 1B. PDCryab1 protect cells against DOX-induced cell damage, as evidenced by increased cell viability and decreased LDH release (Figures 7(c) and 7(d)). To study the function of PDCryab1 in DOX-induced cardiotoxicity, the apoptosis and ROS production levels were evaluated. The results showed that PDCryab1 could reduce the activation of PARP and caspase3 (Figure 7(e)). Simultaneously, 20 *μ*M PDCryab1 also decreased the generation of reactive oxygen species (Figure 7(f)). Besides, we also provided data about early apoptosis and PDCryab1 could reduce cell apoptosis rates, as indicated by mitochondrial membrane potential and TUNEL assay (Figures 7(g) and 7(h)). Last, we verified the SOD and MDA content in the supernatant. Our results showed that PDCryab1 significantly alleviated oxidative stress, as evidenced by increased SOD and decreased MDA content (Figure 7(i)).

### 3.7. Functional Analysis of PDCryab1 In Vivo

**Figure 8:**
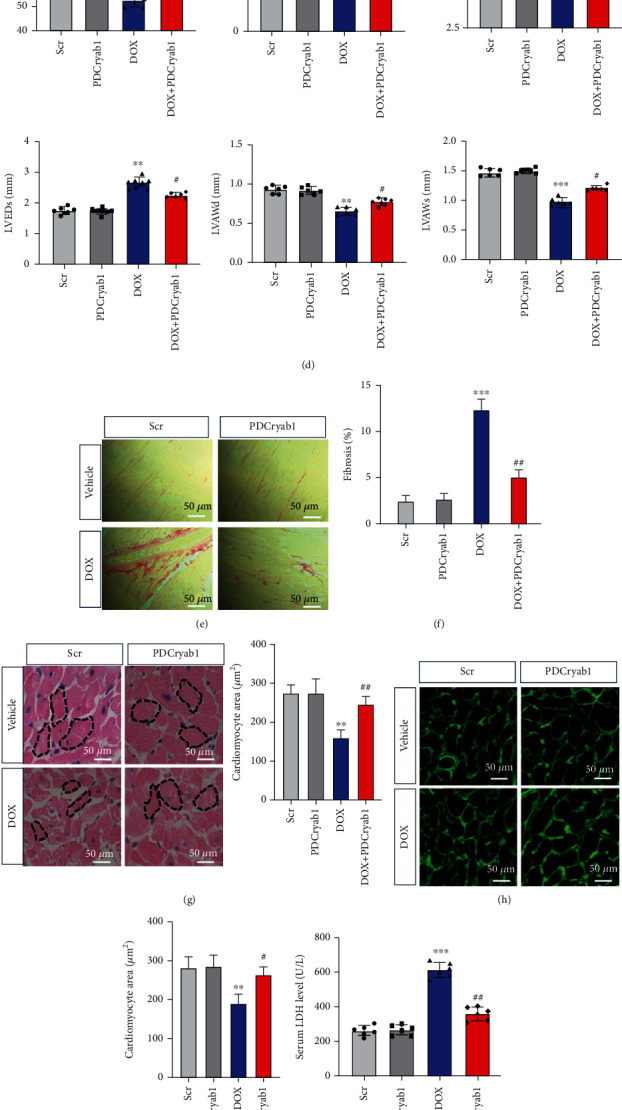
Functional analysis of PDCryab1 in vivo. (a) Schematic map of DOX-induced cardiotoxicity. (b) Body weight was detected (two-way ANOVA analysis with Bonferroni's multiple comparison test). (c) Representative photographs of echocardiography analysis were recorded. (d) Quantification data of echocardiography analysis (two-way ANOVA analysis with Bonferroni's multiple comparison test). (e) Representative photographs of Sirius red staining. (f) Quantification data of Sirius red staining (two-way ANOVA analysis with Bonferroni's multiple comparison test). (g) Representative photographs of HE staining and quantification data of HE staining (two-way ANOVA analysis with Bonferroni's multiple comparison test). (h) Representative photographs of WGA staining. (i) Quantification data of WGA staining (two-way ANOVA analysis with Bonferroni's multiple comparison test). (j) LDH release was detected (two-way ANOVA analysis with Bonferroni's multiple comparison test). (k, l) mRNA levels of ANF and BNP were detected (two-way ANOVA analysis with Bonferroni's multiple comparison test). ^∗∗^*P* < 0.01 and ^∗∗∗^*P* < 0.001 versus the control group. ^#^*P* < 0.05 and ^##^*P* < 0.01 versus the DOX/DOX+Scr group. ns: not statistically significant.

To further investigate the function of PDCryab1, we established a DOX-induced cardiotoxicity model. A cumulative dose of 20 mg/kg of doxorubicin (DOX) was administered via 4 weekly i.p. injections (Figure 8(a)). Body weight was significantly decreased in the DOX injection group, whereas PDCryab1 abolished this effect during DOX injection (Figure 8(b)). Treatment of PDCryab1 significantly improved the cardiac function, as evidenced by echocardiography analysis (Figures 8(c) and 8(d)). We also performed Sirius red staining, and our results showed that treatment of PDCryab1 alleviated DOX-induced cardiac fibrosis (Figures 8(e) and 8(f)). Besides, we verified the cardiomyocyte area via HE staining and WGA staining. Our results demonstrated that intervention of PDCryab1 improved the DOX-induced cardiac damage, as evidenced by increased cardiomyocyte area and decreased LDH release (Figures 8(g)–8(j)). Lastly, heart tissues were harvested to verify the cardiac marker, ANF and BNP. Our results revealed that treatment of PDCryab1 significantly reduced the mRNA level of ANF and BNP, suggesting a beneficial effect of PDCryab1 (Figures 8(k) and 8(l)).

## 4. Discussion

As we all know, DOX is widely used in the treatment of various tumors as a basic chemotherapy drug. However, the cardiotoxicity induced by DOX has become an increasingly serious problem and has been challenging many experts in the cardiovascular field [27]. Although there have been many studies on DOX-induced cardiotoxicity in recent years, the problem has not been resolved. To date, we used peptidomics to comprehensively analyze the changes in peptide profiles related to DOX-induced cardiotoxicity and successfully identified differentially expressed peptides in heart tissues. By analyzing the physicochemical properties and bioinformatics of these differentially expressed peptides, we provide new insights into the clinical problem of DOX-induced cardiotoxicity.

In this study, we identified a total of 236 peptides expressed at a difference that exceeds 2-fold changes. These peptides comprised fewer than 25 amino acids, and the molecular weight was less than 3.0 kDa, which suggested that the peptides identified in this study were valid. Many of these peptides originated from the same precursor protein, which attracted our attention. It is known that most peptides are produced by protein cleavage, and proteases play a key role in the cleavage process by specifically identifying cleavage sites [28]. In addition, the different cleavage sites recognized by the protease will have a great influence on the biological function of the cleaved peptides [29]. Our finding also indicated that the protease follows specific rules in the process of protein cleavage. Physicochemical properties, including peptide length, molecular weight, isoelectric point, and cleavage sites, are helpful for us to select the potential peptides. First, the liposoluble peptides are easily entered cells. Second, the peptides that have a long half-life are stable in cells. Third, relative lower length peptides are easily entered cells.

Through a bioinformatics analysis of these differential peptides, the cellular components enriched with these peptides were the mitochondrial inner membrane and mitochondrial respiratory chain, and the biological functions enriched with these peptides were related to the synthesis and metabolism of ATP. Mitochondria are considered the main target organelles of DOX in cardiomyocytes [30]. Some studies have shown that DOX preferentially accumulates in the mitochondria of cardiomyocytes, causing mitochondrial swelling and mitochondrial dysfunction [31, 32]. The pathway analysis results show that these peptides are mainly involved in oxidative phosphorylation and metabolic pathways. In energy metabolism, ATP is the main energy supplying compound in the body, and the main mechanism of its formation is oxidative phosphorylation. The decrease in mitochondrial energy supply caused by DOX can lead to a change in cardiac metabolism. The levels of ATP and creatine phosphate in the hearts of DOX-treated rats were decreased, indicating a decrease in mitochondrial energy metabolism [33]. In addition, DOX can inhibit the use of glucose by the myocardium while reducing the beta-oxidation of long-chain fatty acids, which may eventually lead to the development of myocardial energy metabolism disorders [34]. Therefore, attenuating the myocardial metabolic changes caused by DOX is one of the strategies to alleviate DOX-induced cardiotoxicity and in which these peptides may play key roles.

Cardiomyocyte apoptosis is a vital biological event of DOX-induced cardiotoxicity [4]. Studies have found that some biologically active peptides, such as ICL1-9 [35] and pNaKtide [4, 26], play a protective role in the process of cardiomyocyte apoptosis. In this study, many precursor proteins of differentially expressed peptides are involved in the regulation of cardiomyocyte apoptosis, including heat shock protein beta-1 (Hspb1) [36], alpha-crystallin B chain (Cryab) [37], heat shock protein beta-6 (Hspb6) [38], and actin, alpha cardiac muscle 1 (Actc1) [39]. Cryab is the most abundant small heat shock protein (sHSP) in cardiomyocytes, and it can antagonize myocardial ischemia/reperfusion injury and is essential for normal cardiac function [40]. In addition, Cryab can inhibit the apoptosis of neonatal mouse cardiomyocytes treated with H_2_O_2_ [37]. Some studies have shown that peptides often play a biological role similar to that of their precursor proteins [41]. As shown in Table 1, of the 31 peptides that we predicted to be active, 6 were from Cryab, and all of them were downregulated in the DOX treatment group, findings consistent with the theory stated above. Therefore, we speculate that these six peptides may be involved in the regulation of Cryab in cardiomyocytes and may have the same function as Cryab. Interestingly, the peptides derived from Hspb1 are both upregulated and downregulated, while Hspb1 is recognized as a protein with a cardioprotective effect. The function of these peptides is worthy of further verification. If the upregulated peptides also have cardioprotective effects, whether the peptide has the same function as its precursor protein needs to be further clarified.

In this study, PDCryab1 (SPFYLRPPSF) was a downregulated peptide in the DOX treatment group that was derived from the Cryab protein, has high homology among various species, and had not been previously reported. Our previous experiments demonstrated that PDCryab1 can inhibit cardiomyocyte apoptosis, reduce the production of reactive oxygen species, improve cardiac function, and ameliorate myocardial fibrosis. Although we have confirmed that PDCryab1 has a myocardial protective effect in vitro and in vivo, there were still some limitations to our study. For example, whether the type of cleavage or modification affects the function of PDCryab1 remains to be verified. In addition, the specific mechanism by which PDCryab1 exerts its biological function is also particularly important and will be the focus of our future research.

The peptide AEGPAAVTLAAPAFSRALNRQL was downregulated in the DOX treatment group. It was in the sHSP domain and interaction with the TGFB1I1 region of the Hspb1 protein. Hspb1 can inhibit the apoptosis caused by oxidative stress and protect the myocardium [36]. The domain is a region in a protein that has an independent structure and function, and this function often does not depend on the other regions of a protein molecule. Therefore, a peptide located in a domain region is more likely to have independent biological activity [42]. AEGPAAVTLAAPAFSRALNRQL was in the domain of Hspb1, and its predicted biological activity score was 0.59 (more than 0.5), which suggested that it may have an antiapoptotic effect and may be another therapeutic target of DOX-induced cardiotoxicity. In addition, Hspb1 can interact with VEGF and transforming growth factor (TGFB1I1) to regulate angiogenesis [43], and this peptide is in the region that interacts with TGFB1I1. We speculate that this peptide may also play a previously unidentified role in angiogenesis.

A peptide derived from Hmgb1 also attracted our attention. Its sequence was DPNAPKRPPSA (91-101). High mobility group box 1 (Hmgb1) is a DNA-binding nuclear nonhistone protein that plays an important role in the occurrence and development of cardiovascular diseases [44]. In general, Hmgb1 is passively released from necrotic cells, and living cells can actively secrete it under certain pathological conditions [45]. Studies have shown that DOX can significantly increase the expression level of Hmgb1 in cardiomyocytes, resulting in cardiomyocyte apoptosis and cardiac dysfunction, and that silencing Hmgb1 can protect the myocardium from DOX-induced cardiotoxicity [45, 46]. In addition, Hmgb1 has also been proven to be involved in DOX-induced autophagy-related cardiotoxicity and is predicted to be a biomarker of DOX-induced cardiotoxicity [47]. However, a recent study showed that Hmgb1 can upregulate the expression of Hspb1 and attenuate the cardiomyocyte apoptosis associated with DOX-induced cardiomyopathy [48]. Therefore, Hmgb1 undoubtedly plays an important role in DOX-induced cardiomyocyte apoptosis, but the specific effect of Hmgb1 on cardiomyocyte apoptosis remains to be clarified. Here, we found a peptide derived from Hmgb1 that was downregulated in the DOX treatment group and had a high prediction score for biological activity. This peptide was located in the region with cytokine-stimulating activity and a phosphorylation site (100). The elucidation of the function of this peptide will help to not only clarify the specific effect of Hmgb1 on cardiomyocyte apoptosis but also provide a new intervention strategy for DOX-induced cardiotoxicity.

In summary, we used peptidomics to elucidate the mechanism of DOX-induced cardiotoxicity and explore cardiotoxicity protection strategies; 236 differentially expressed peptides were successfully screened in this study. Through bioinformatics analysis and experimental verification, PDCryab1 became a candidate for protecting the myocardium against DOX-induced cell apoptosis. Our study provides a new approach for the treatment of DOX-induced cardiotoxicity.

## Figures and Tables

**Table 1:** The details of 31 predicted peptides which may have cardioprotective function.

Protein	UniProt accession	Peptide sequence	Peptide location	PTM description (positions)	Domain/region description	Peptide ranker score	Fold change
Atp5pb	Q9CQQ7	PLPPLPEYGGKVRLGLIPEEF	43-63			0.64	-13.77
Cox2	P00405	EMVPLKYFENWSASMI	212-227			0.61	-40.55
Cox6b	P56391	SAWDDRIAEGTFPGK	71-85		CHCH	0.84	-20.82
Cox6a2	P43023	ASAAKGDHGGAGANTWRLLT	13-32		Topological domain	0.84	-20.82
Cox6a2	P43023	KPFAWGDGNHTL	70-81		Topological domain	0.69	-12.77
Cox6a2	P43023	SAAKGDHGGAGANTWRLLTF	14-33			0.85	-5.96
Cox6a2	P43023	SAAKGDHGGAGANTWRLLTFVL	14-35			0.64	-47.59
Cox6a2	P43023	TKPFAWGDGNHTLF	69-82		Topological domain	0.71	-3.70
Cryab	P23927	FGEHLLESDLF	28-38			0.53	-24.10
Cryab	P23927	LFDQFFGEH	23-31			0.62	-9.15
Cryab	P23927	SPFYLRPPSF	45-54	Phosphoserine (45), omega-N-methylated arginine (50)		0.92	-2.69
Cryab	P23927	SPFYLRPPSFLR	45-56	Phosphoserine (45), omega-N-methylated arginine (50)		0.92	-9.66
Cryab	P23927	SPFYLRPPSFLRAPS	45-59	Phosphoserine (45, 59), omega-N-methylated arginine (50)	sHSP	0.81	-9.10
Cryab	P23927	TSLSPFYLRPPSFL	42-55	Phosphoserine (45), omega-N-methylated arginine (50)		0.76	-6.90
Cs	Q9CZU6	SFAAAMNGLAGPLHGLANQEV	288-308			0.61	-3.87
Cycs	P62897	LENPKKYIPGTKMIF	69-83	N6-Acetyllysine (73), N6-succinyllysine (73)	Iron (heme axial ligand)	0.66	-20.24
Fabp3	P11404	VDSKNFDDYMKSLGVGF	12-28	Phosphotyrosine; by Tyr-kinases (20), phosphoserine (23)		0.51	-12.80
Fabp3	P11404	ADAFVGTWKLVDSKNF	2-17	N-Acetylalanine (2), phosphothreonine (8)		0.58	-8.14
Hba	P01942	FASFPTTKT	34-42			0.53	-9.31
Hmgb1	P63158	DPNAPKRPPSA	91-101	Phosphoserine (100)	HMG box 2, LPS binding (lipid A), cytokine-stimulating activity	0.53	-8.52
Hspe1	Q64433	AGQAFRKFLPLFDRVL	2-17	N-Acetylalanine (2), N6-acetyllysine (8)		0.87	-51.60
Hspb1	P14602	AEGPAAVTLAAPAFSRALNRQL	64-85		sHSP, interaction with TGFB1I1	0.59	-7.88
Hspb1	P14602	APAFSRAL	74-81		Interaction with TGFB1I1	0.77	293.23
Hspb1	P14602	PSWEPFRDWYPAHS	14-27	Phosphoserine; by MAPKAPK2, MAPKAPK3, PKA, and PKC (15, 27)		0.75	-4.05
Hspb6	Q5EBG6	ASAPLPGFSAPGRLFDQ	15-31	Phosphoserine (16)	Involved in stabilization of the HSPB1 : HSBP6 heterodimer	0.82	-13.42
Ldb3	Q9JKS4	PRGAPAYNPTGPQVTPLARGTFQRA	511-535	Omega-N-methylarginine (512, 529)		0.53	-12.84
Ldb3	Q9JKS4	SGPVLPLGSPVAK	156-168			0.61	-7.08
Myl2	P51667	KEAPGPINFTVF	71-82			0.77	-14.90
Ndufs4	Q9CXZ1	ASTADPLSNMVLTF	115-128			0.63	-45.08
Ndufs5	Q99LY9	PFLDIQKKLGISLDR	2-16			0.58	-7.48
Uqcrfs1	Q9CR68	GVAGALRPLLQGAVPAASEPPVLDV	21-45			0.81	-12.83

## Data Availability

The data that support the findings of this study are available from the corresponding author upon reasonable request.

## Supplementary Materials

Supplementary Materials Supplemental Fig. 1: conservation analysis and HCD MS/MS annotation of the peptide PDCryab1. (A) Conservation analysis of PDCryab1 (SPFYLRPPSF) in various species. (B) HCD MS/MS annotation of the peptide PDCryab1 (SPFYLRPPSF) derived from Cryab spanning amino acids 45-54. All fragment ions are annotated to within 20 ppm. Supple. Table 1: 236 differentially expressed peptides and precursor proteins (DOX vs. control). Supple. Table 2: identification of unique peptides. Supple. Table 3: the same peptides derived from several precursor proteins.

## References

[1] Damiani R. M., Moura D. J., Viau C. M., Caceres R. A., Henriques J. A. P., Saffi J. (2016). Pathways of cardiac toxicity: comparison between chemotherapeutic drugs doxorubicin and mitoxantrone. *Archives of Toxicology*.

[2] Cagel M., Grotz E., Bernabeu E., Moretton M. A., Chiappetta D. A. (2017). Doxorubicin: nanotechnological overviews from bench to bedside. *Drug Discovery Today*.

[3] Li D. L., Hill J. A. (2014). Cardiomyocyte autophagy and cancer chemotherapy. *Journal of Molecular and Cellular Cardiology*.

[4] Shi J., Abdelwahid E., Wei L. (2011). Apoptosis in anthracycline cardiomyopathy. *Current Pediatric Reviews*.

[5] Rochette L., Guenancia C., Gudjoncik A. (2015). Anthracyclines/trastuzumab: new aspects of cardiotoxicity and molecular mechanisms. *Trends in Pharmacological Sciences*.

[6] Wallace K. B. (2007). Adriamycin-induced interference with cardiac mitochondrial calcium homeostasis. *Cardiovascular Toxicology*.

[7] Chen C., Jiang L., Zhang M. (2019). Isodunnianol alleviates doxorubicin-induced myocardial injury by activating protective autophagy. *Food & Function*.

[8] Dallas D. C., Guerrero A., Parker E. A. (2015). Current peptidomics: applications, purification, identification, quantification, and functional analysis. *Proteomics*.

[9] Slavoff S. A., Mitchell A. J., Schwaid A. G. (2013). Peptidomic discovery of short open reading frame–encoded peptides in human cells. *Nature Chemical Biology*.

[10] Rubakhin S. S., Churchill J. D., Greenough W. T., Sweedler J. V. (2006). Profiling signaling peptides in single mammalian cells using mass spectrometry. *Analytical Chemistry*.

[11] Dang L. T., Feric N. T., Laschinger C. (2014). Inhibition of apoptosis in human induced pluripotent stem cells during expansion in a defined culture using angiopoietin-1 derived peptide QHREDGS. *Biomaterials*.

[12] Chimen M., McGettrick H. M., Apta B. (2015). Homeostatic regulation of T cell trafficking by a B cell-derived peptide is impaired in autoimmune and chronic inflammatory disease. *Nature Medicine*.

[13] Chaturvedi L. S., Basson M. D. (2013). Glucagonlike peptide 2 analogue teduglutide: stimulation of proliferation but reduction of differentiation in human Caco-2 intestinal epithelial cells. *JAMA Surgery*.

[14] Zhu H., Wang X., Wallack M. (2015). Intraperitoneal injection of the pancreatic peptide amylin potently reduces behavioral impairment and brain amyloid pathology in murine models of Alzheimer’s disease. *Molecular Psychiatry*.

[15] Ivell R., Anand-Ivell R. (2018). Insulin-like peptide 3 (INSL3) is a major regulator of female reproductive physiology. *Human Reproduction Update*.

[16] Fosgerau K., Hoffmann T. (2015). Peptide therapeutics: current status and future directions. *Drug Discovery Today*.

[17] Voigt A., Jelinek H. F. (2016). Humanin: a mitochondrial signaling peptide as a biomarker for impaired fasting glucose-related oxidative stress. *Physiological Reports*.

[18] Thummasorn S., Shinlapawittayatorn K., Chattipakorn S. C., Chattipakorn N. (2017). High-dose humanin analogue applied during ischemia exerts cardioprotection against ischemia/reperfusion injury by reducing mitochondrial dysfunction. *Cardiovascular Therapeutics*.

[19] Lue Y., Gao C., Swerdloff R. (2018). Humanin analog enhances the protective effect of dexrazoxane against doxorubicin-induced cardiotoxicity. *American Journal of Physiology-Heart and Circulatory Physiology*.

[20] Lee K. H., Cho H., Lee S. (2017). Enhanced-autophagy by exenatide mitigates doxorubicin-induced cardiotoxicity. *International Journal of Cardiology*.

[21] Pchejetski D., Foussal C., Alfarano C. (2012). Apelin prevents cardiac fibroblast activation and collagen production through inhibition of sphingosine kinase 1. *European Heart Journal*.

[22] Oh J., Lee B. S., Lim G. (2020). Atorvastatin protects cardiomyocyte from doxorubicin toxicity by modulating survivin expression through FOXO1 inhibition. *Journal of Molecular and Cellular Cardiology*.

[23] Mooney C., Haslam N. J., Pollastri G., Shields D. C. (2012). Towards the improved discovery and design of functional peptides: common features of diverse classes permit generalized prediction of bioactivity. *PLoS One*.

[24] Montone C. M., Capriotti A. L., Cavaliere C. (2018). Peptidomic strategy for purification and identification of potential ACE-inhibitory and antioxidant peptides in Tetradesmus obliquus microalgae. *Analytical and Bioanalytical Chemistry*.

[25] Lee F. Y., Shao P. L., Wallace C. G. (2018). Combined therapy with SS31 and mitochondria mitigates myocardial ischemia-reperfusion injury in rats. *International Journal of Molecular Sciences*.

[26] Li H., Yin A., Cheng Z. (2018). Attenuation of Na/K-ATPase/Src/ROS amplification signal pathway with pNaktide ameliorates myocardial ischemia-reperfusion injury. *International Journal of Biological Macromolecules*.

[27] Liu D., Ma Z., Di S. (2018). AMPK/PGC1*α* activation by melatonin attenuates acute doxorubicin cardiotoxicity via alleviating mitochondrial oxidative damage and apoptosis. *Free Radical Biology and Medicine*.

[28] Li F., Wang Y., Li C. (2019). Twenty years of bioinformatics research for protease-specific substrate and cleavage site prediction: a comprehensive revisit and benchmarking of existing methods. *Briefings in Bioinformatics*.

[29] van den Berg B. H. J., Tholey A. (2012). Mass spectrometry-based proteomics strategies for protease cleavage site identification. *Proteomics*.

[30] Ichikawa Y., Ghanefar M., Bayeva M. (2014). Cardiotoxicity of doxorubicin is mediated through mitochondrial iron accumulation. *Journal of Clinical Investigation*.

[31] Wang P., Wang L., Lu J. (2019). SESN2 protects against doxorubicin-induced cardiomyopathy via rescuing mitophagy and improving mitochondrial function. *Journal of Molecular and Cellular Cardiology*.

[32] Sardão V. A., Oliveira P. J., Holy J., Oliveira C. R., Wallace K. B. (2009). Morphological alterations induced by doxorubicin on H9c2 myoblasts: nuclear, mitochondrial, and cytoskeletal targets. *Cell Biology and Toxicology*.

[33] Tokarska-Schlattner M., Zaugg M., Zuppinger C., Wallimann T., Schlattner U. (2006). New insights into doxorubicin-induced cardiotoxicity: the critical role of cellular energetics. *Journal of Molecular and Cellular Cardiology*.

[34] Carvalho R. A., Sousa R. P., Cadete V. J. (2010). Metabolic remodeling associated with subchronic doxorubicin cardiomyopathy. *Toxicology*.

[35] Grisanti L. A., Thomas T. P., Carter R. L. (2018). Pepducin-mediated cardioprotection via beta-arrestin-biased beta2-adrenergic receptor-specific signaling. *Theranostics*.

[36] Liu X., Liu K., Li C. (2019). Heat-shock protein B1 upholds the cytoplasm reduced state to inhibit activation of the Hippo pathway in H9c2 cells. *Journal of Cellular Physiology*.

[37] Chis R., Sharma P., Bousette N. (2012). *α*-Crystallin B prevents apoptosis after H_2_O_2_ exposure in mouse neonatal cardiomyocytes. *American Journal of Physiology-Heart and Circulatory Physiology*.

[38] Liu G. S., Zhu H., Cai W. F. (2018). Regulation of BECN1-mediated autophagy by HSPB6: insights from a human HSPB6(S10F) mutant. *Autophagy*.

[39] Jiang H. K., Qiu G. R., Li-Ling J., Xin N., Sun K. L. (2010). Reduced ACTC1 expression might play a role in the onset of congenital heart disease by inducing cardiomyocyte apoptosis. *Circulation Journal*.

[40] Martin J. L., Mestril R., Hilal-Dandan R., Brunton L. L., Dillmann W. H. (1997). Small heat shock proteins and protection against ischemic injury in cardiac myocytes. *Circulation*.

[41] Gao X., Zhang H., Zhuang W. (2014). PEDF and PEDF-derived peptide 44mer protect cardiomyocytes against hypoxia-induced apoptosis and necroptosis via anti-oxidative effect. *Scientific Reports*.

[42] Pereira J., Lupas A. N. (2018). The ancestral KH peptide at the root of a domain family with three different folds. *Bioinformatics*.

[43] Choi S. H., Lee H. J., Jin Y. B. (2014). MMP9 processing of HSPB1 regulates tumor progression. *PLoS One*.

[44] Funayama A., Shishido T., Netsu S. (2013). Cardiac nuclear high mobility group box 1 prevents the development of cardiac hypertrophy and heart failure. *Cardiovascular Research*.

[45] Yao Y., Xu X., Zhang G., Zhang Y., Qian W., Rui T. (2012). Role of HMGB1 in doxorubicin-induced myocardial apoptosis and its regulation pathway. *Basic Research in Cardiology*.

[46] Taskin E., Guven C., Tunc K. S. (2020). Silencing HMGB1 expression inhibits adriamycin's heart toxicity via TLR4 dependent manner through MAPK signal transduction. *Journal of B.U.O.N.*.

[47] Luo P., Zhu Y., Chen M. (2018). HMGB1 contributes to adriamycin-induced cardiotoxicity via up-regulating autophagy. *Toxicology Letters*.

[48] Narumi T., Shishido T., Otaki Y. (2015). High-mobility group box 1-mediated heat shock protein beta 1 expression attenuates mitochondrial dysfunction and apoptosis. *Journal of Molecular and Cellular Cardiology*.

