# Supplementary material for: Peptidomics Analysis Reveals Peptide PDCryab1 Inhibits Doxorubicin-Induced Cardiotoxicity
Source: Oxid Med Cell Longev. 2020 Oct 13;2020:7182428. doi: 10.1155/2020/7182428 (PMC7582065; doi:10.1155/2020/7182428)
Supplement: Supplementary Materials — Supplemental Fig. 1: conservation analysis and HCD MS/MS annotation of the peptide PDCryab1. (A) Conservation analysis of PDCryab1 (SPFYLRPPSF) in various species. (B) HCD MS/MS annotation of the peptide PDCryab1 (SPFYLRPPSF) derived from Cryab spanning amino acids 45-54. All fragment ions are annotated to within 20 ppm. Supple. Table 1: 236 differentially expressed peptides and precursor proteins (DOX vs. control). Supple. Table 2: identification of unique peptides. Supple. Table 3: the same peptides derived from several precursor proteins. [file 7182428.f1.docx]

**Supplemental Fig.1 Conservation analysis and HCD MS/MS annotation of the peptide PDCryab1**. (A). Conservation analysis of PDCryab1 (SPFYLRPPSF) in various species. (B). HCD MS/MS annotation of the peptide PDCryab1 (SPFYLRPPSF) derived from Cryab spanning amino acids 45-54. All fragment ions are annotated to within 20 ppm.

**Supple.Table 1. 236 differentially expressed peptides and precursor proteins (DOX v.s Control).**

**Supple. Table 2.  Identification of unique peptides.**

**Supple.Table 3. The same peptides derived from several precursor proteins.**

**Supple. Fig 1**


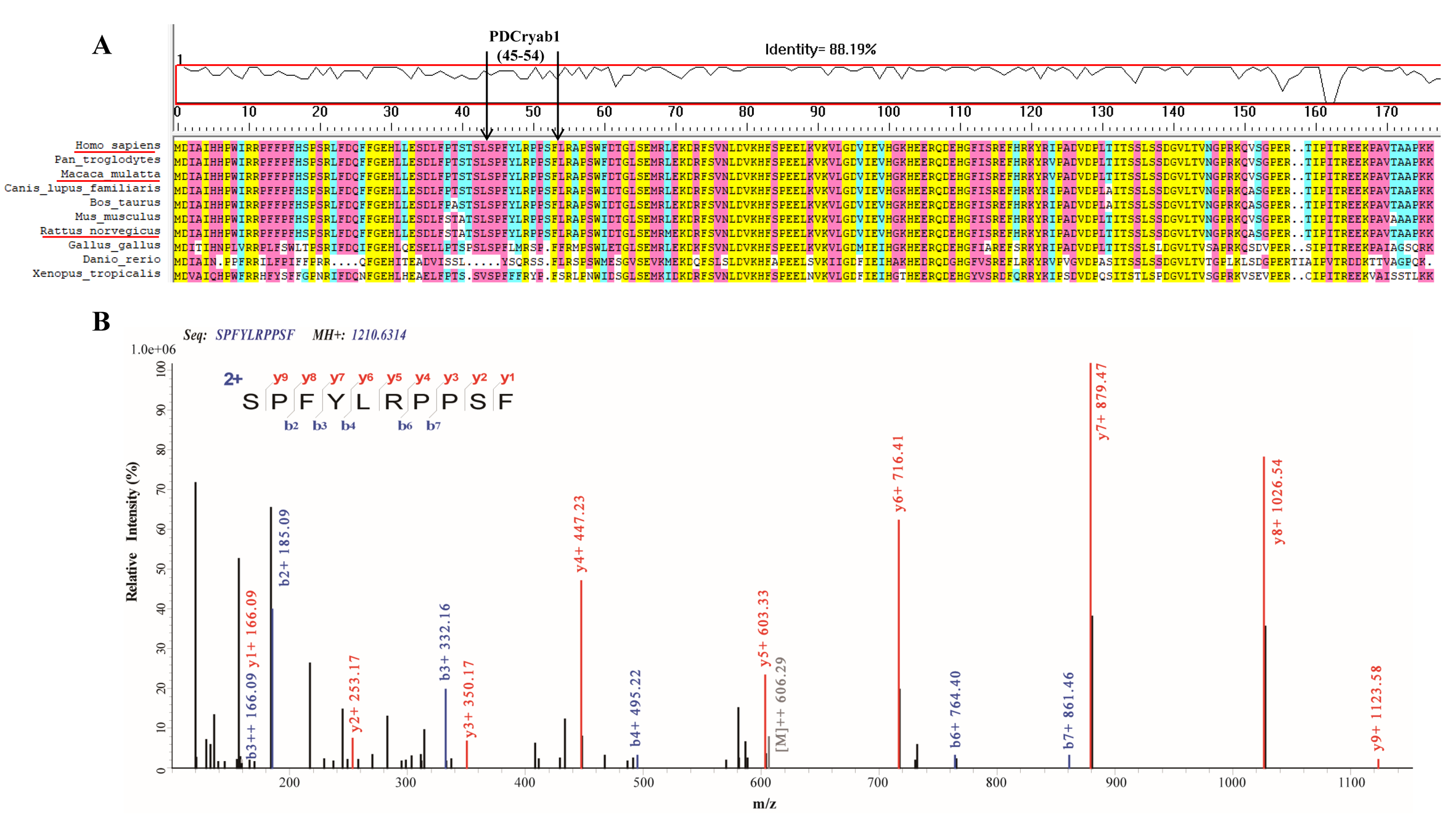


**Supple.Table 1. 236 differentially expressed peptides and precursor proteins (DOX v.s Control)**

| **Uniprot Accession** | **Protein Symbol** | **Protein Name** | **Peptide Sequence** | **Fold Change** | **p Value** |
| --- | --- | --- | --- | --- | --- |
| O08749 | DLDH_MOUSE | Dihydrolipoyl dehydrogenase | ADTDGMVKILG | -16.60 | 0.004619851 |
|  |  |  | EANLAAAFGKPIN | -4.12 | 0.006752887 |
|  |  |  | STDRVLGAHILGPGAGEMVNE | -13.01 | 0.007023017 |
| O35943 | FRDA_MOUSE | Frataxin | DLSSLAYSGKGT | -11.52 | 0.008995314 |
|  |  |  | LNTKLDLSSLAYSGKGT | -10.14 | 0.006904108 |
| O54724 | CAVN1_MOUSE | Caveolae-associated protein 1 | MEDVTLHIVER | -7.86 | 0.002295846 |
| O70251 | EF1B_MOUSE | Elongation factor 1-beta | GFGDLKTPAGLQVLNDYL | -15.39 | 0.001600072 |
| O70433 | FHL2_MOUSE | Four and a half LIM domains protein 2 | KPITTGGVTY | -10.85 | 0.000191147 |
| P00405 | COX2_MOUSE | Cytochrome c oxidase subunit 2 | EMVPLKYFENWSASMI | -40.55 | 0.031589525 |
| P00848 | ATP6_MOUSE | F-ATPase protein 6 | LLPHTFTPTTQLSMN | -3.51 | 0.02746919 |
| P01942 | HBA_MOUSE | Hemoglobin subunit alpha | ASFPTTKTYFPHFDV | -8.41 | 0.00313127 |
|  |  |  | ASFPTTKTYFPHFDVS | -16.31 | 0.007865059 |
|  |  |  | ASLDKFLASVSTVLTSK | -5.15 | 0.007888078 |
|  |  |  | AWGKIGGHGAEYGAEAL | -24.27 | 0.02020496 |
|  |  |  | FASFPTTKT | -9.31 | 0.001159195 |
|  |  |  | FASFPTTKTYFPH | -15.62 | 0.002613689 |
|  |  |  | LASVSTVLTSKY | -18.26 | 0.001554873 |
| P03911 | NU4M_MOUSE | NADH-ubiquinone oxidoreductase chain 4 | LTTSPKLITGLTM | -51.93 | 0.004830935 |
| P03921 | NU5M_MOUSE | NADH-ubiquinone oxidoreductase chain 5 | QFGLHPWLPSAM | 4.55 | 0.009982783 |
| P04247 | MYG_MOUSE | Myoglobin | RNDIAAKY | -5.30 | 0.042523781 |
| P05064 | ALDOA_MOUSE | Fructose-bisphosphate aldolase A | GEHTPSALAI | -14.87 | 0.000533499 |
|  |  |  | KVDKGVVPL | -20.29 | 0.009154271 |
|  |  |  | MENANVLARY | -2.47 | 0.041512038 |
| P05201 | AATC_MOUSE | Aspartate aminotransferase, cytoplasmic | APPSVFAQVPQAPPVLVFKL | -10.92 | 0.004514268 |
| P05202 | AATM_MOUSE | Aspartate aminotransferase, mitochondrial | RDAGMQLQGYR | -2.17 | 0.033802621 |
| P05202 | AATM_MOUSE | Aspartate aminotransferase, mitochondrial | SNPPLNGARIAATIL | -13.42 | 0.00119507 |
| P06151 | LDHA_MOUSE | L-lactate dehydrogenase A chain | ATLKDQLIVNL | -12.51 | 1.75141E-05 |
| P07724 | ALBU_MOUSE | Serum albumin | NDLGEQHFKGLVLIA | -9.95 | 0.013334502 |
| P08249 | MDHM_MOUSE | Malate dehydrogenase, mitochondrial | AKVAVLGASGGIGQPLSLL | -14.38 | 0.001624339 |
|  |  |  | TLYDIAHTPGVAADL | -5.30 | 0.004575909 |
|  |  |  | VLGASGGIGQPLSLLLKNSP | -3.41 | 0.000462285 |
| P09542 | MYL3_MOUSE | Myosin light chain 3 | ALGQNPTQAEV | -5.15 | 0.04071125 |
|  |  |  | LLFDRTPKGEMKITY | -35.80 | 0.026682708 |
|  |  |  | MDFETFLPMLQHISKN | -4.85 | 2.23187E-05 |
|  |  |  | NPTQAEVLRV | -6.02 | 0.001530228 |
|  |  |  | NSKMMDFETFLPMLQH | -11.74 | 0.000848178 |
|  |  |  | TPEQIEEFKEAF | -14.75 | 0.008229135 |
| P09813 | APOA2_MOUSE | Apolipoprotein A-II | FSSLMNLEEKPAPAA | -3.07 | 0.015245298 |
|  |  |  | MNLEEKPAPAA | -15.31 | 0.015915615 |
| P0DN34 | NDUB1_MOUSE | NADH dehydrogenase 1 beta subcomplex subunit 1 | ELRPNEEVTWK | -9.16 | 0.003905328 |
| P11404 | FABPH_MOUSE | Fatty acid-binding protein, heart | ADAFVGTWKLVDSKNF | -8.14 | 0.000619922 |
| P11404 | FABPH_MOUSE | Fatty acid-binding protein, heart | VDSKNFDDYMKSLGVGF | -12.80 | 0.009574516 |
| P12787 | COX5A_MOUSE | Cytochrome c oxidase subunit 5A | QELRPTLNELGISTPEEL | -6.02 | 0.007356006 |
|  |  |  | RKGMNTLVGY | 2.80 | 0.013686884 |
|  |  |  | RKGMNTLVGYDL | 6.12 | 0.020577892 |
|  |  |  | RKGMNTLVGYDLVPEPKIIDAALRA | 3.84 | 0.007848727 |
|  |  |  | RPTLNELGISTPEELGLDKV | 2.43 | 0.001652257 |
|  |  |  | RRLNDFASAVRILEV | 3.75 | 0.047441106 |
|  |  |  | VIQELRPTLNEL | -12.67 | 0.002039973 |
| P14602 | HSPB1_MOUSE | Heat shock protein beta-1 | AEGPAAVTLAAPAFSRAL | 4.97 | 0.02797717 |
|  |  |  | AEGPAAVTLAAPAFSRALNRQL | -7.88 | 0.007311024 |
|  |  |  | APAFSRAL | 293.23 | 3.64367E-07 |
|  |  |  | GWPGYVRPLPAATAEGPAAVTL | -28.52 | 0.002273303 |
|  |  |  | PSWEPFRDWYPAHS | -4.05 | 8.28669E-05 |
|  |  |  | SLSPEGTLTVEAPLPKAVTQ | -7.11 | 0.005430448 |
| P16858 | G3P_MOUSE | Glyceraldehyde-3-phosphate dehydrogenase | SWYDNEYGYSNRVVDL | -10.69 | 0.008017504 |
|  |  |  | YDNEYGYSNR | -3.78 | 0.012392022 |
| P17095 | HMGA1_MOUSE | High mobility group protein HMG-I/HMG-Y | SESGSKSSQPL | -20.00 | 0.002629708 |
| P17742 | PPIA_MOUSE | Peptidyl-prolyl cis-trans isomerase A | DITADDEPLGRVSF | -12.33 | 0.007458417 |
|  |  |  | FDITADDEPLGRVS | -4.57 | 0.002761553 |
|  |  |  | FDITADDEPLGRVSFEL | -33.67 | 0.005239318 |
| P17751 | TPIS_MOUSE | Triosephosphate isomerase | APPTAYIDFA | -16.49 | 0.000937944 |
|  |  |  | GASLKPEFVDIINAKQ | -3.92 | 0.013853559 |
| P20152 | VIME_MOUSE | Vimentin | FGGSGTSSRP | -3.15 | 0.034950291 |
|  |  |  | FGGSGTSSRPSS | -4.26 | 0.043299188 |
|  |  |  | STRSVSSSSY | -4.03 | 0.048750828 |
| P21812 | MCPT4_MOUSE | Mast cell protease 4 | GRGDAKPPAVFTR | -5.82 | 0.006586018 |
| P23927 | CRYAB_MOUSE | Alpha-crystallin B chain | EKDRFSVNL | -16.34 | 0.001420864 |
|  |  |  | FGEHLLESDLF | -24.10 | 0.001271244 |
|  |  |  | FGEHLLESDLFST | 3.90 | 0.04501029 |
|  |  |  | LFDQFFGEH | -9.15 | 0.001396962 |
|  |  |  | SLSSDGVLTVNGPRKQ | -3.64 | 0.04103295 |
|  |  |  | SPFYLRPPSF | -2.69 | 0.043427747 |
|  |  |  | SPFYLRPPSFLR | -9.66 | 0.003278627 |
|  |  |  | SPFYLRPPSFLRAPS | -9.10 | 0.000315885 |
|  |  |  | TSLSPFYLRPPSFL | -6.90 | 0.005148938 |
| P26350 | PTMA_MOUSE | Prothymosin alpha | SDAAVDTSSEITTKDLK | -4.37 | 0.000806074 |
| P27546 | MAP4_MOUSE | Microtubule-associated protein 4 | TTPTVSKATSPSTLVSTGPSSR | -20.00 | 0.004056981 |
| P32020 | NLTP_MOUSE | Non-specific lipid-transfer protein | LQNLQLQPGKAKL | -17.40 | 0.02357448 |
|  |  |  | QNLQLQPGKAK | -12.76 | 0.00025855 |
| P34884 | MIF_MOUSE | Macrophage migration inhibitory factor | PMFIVNTNVPRASVPEGFLSEL | -14.91 | 0.000414654 |
| P43023 | CX6A2_MOUSE | Cytochrome c oxidase subunit 6A2 | ASAAKGDHGGAGANTWRLLT | -20.82 | 0.017439051 |
|  |  |  | KPFAWGDGNHTL | -12.77 | 0.006350581 |
|  |  |  | RLLTFVLALPGVA | 67.65 | 0.000890047 |
|  |  |  | SAAKGDHGGAGANTWRLLTF | -5.96 | 0.005534812 |
|  |  |  | SAAKGDHGGAGANTWRLLTFVL | -47.59 | 0.000812834 |
|  |  |  | TKPFAWGDGNHTLF | -3.70 | 0.036156342 |
| P43274 | H14_MOUSE | Histone H1.4 | SETAPAAPAAPAPAEK | -8.29 | 0.042299634 |
| P48024 | EIF1_MOUSE | Eukaryotic translation initiation factor 1 | SAIQNLHSFDPF | -5.10 | 0.024612902 |
| P48772 | COX8B_MOUSE | Cytochrome c oxidase subunit 8B | MVPAGWVLAHLESYKKSSAA | -11.27 | 0.031405242 |
|  |  |  | VSAKPAKTPTSAVEQAVGISA | -96.79 | 0.030297185 |
| P48787 | TNNI3_MOUSE | Troponin I | IDALSGMEGRK | -9.62 | 0.017961102 |
|  |  |  | SADAMMQALLGTRA | -5.74 | 0.01134573 |
| P50462 | CSRP3_MOUSE | Cysteine and glycine-rich protein 3 | FGPTGIGFGGLTQQVEKKE | -4.10 | 0.005537236 |
|  |  |  | GGLTQQVEKKE | -4.25 | 0.04104828 |
| P50752 | TNNT2_MOUSE | Troponin T, cardiac muscle | QQKYEINVL | -93.46 | 0.01138768 |
| P51667 | MLRV_MOUSE | Myosin regulatory light chain 2 | GRVNVKNEEIDEMIKEAPGPINF | -22.71 | 0.000968769 |
|  |  |  | KEAPGPINFTVF | -14.90 | 0.001038784 |
|  |  |  | LTMFGEKLKGADPEETILNA | -35.91 | 1.76504E-05 |
|  |  |  | VKNEEIDEMIKEAPGPINF | -23.58 | 0.019131495 |
|  |  |  | VKNEEIDEMIKEAPGPINFT | -34.95 | 8.3462E-06 |
| P56391 | CX6B1_MOUSE | Cytochrome c oxidase subunit 6B1 | SAWDDRIAEGTFPGK | -3.34 | 0.021835116 |
| P56393 | COX7B_MOUSE | Cytochrome c oxidase subunit 7B | TQIGIEWNMSPVGRVTPKEWRDQ | -7.71 | 0.004276151 |
| P56480 | ATPB_MOUSE | ATP synthase subunit beta | AAQASAAPKAGTATG | -8.67 | 0.008738578 |
| P57780 | ACTN4_MOUSE | Alpha-actinin-4 | VDYHAANQAYQYGP | -3.21 | 0.001077209 |
| P62631 | EF1A2_MOUSE | Elongation factor 1-alpha 2 | TSQVIILNHPGQISAG | -7.61 | 5.9004E-06 |
| P62827 | RAN_MOUSE | GTP-binding nuclear protein Ran | VATLGVEVHPL | -11.89 | 0.009045927 |
| P62897 | CYC_MOUSE | Cytochrome c, somatic | LENPKKYIPGTKMIF | -20.24 | 0.007796137 |
|  |  |  | MEYLENPKKYIPGTK | -27.71 | 0.002408031 |
|  |  |  | TDANKNKGITWGEDT | -48.96 | 0.017611042 |
|  |  |  | TDANKNKGITWGEDTL | -2.20 | 0.042894216 |
|  |  |  | TDANKNKGITWGEDTLME | -171.25 | 0.014828294 |
| P63030 | MPC1_MOUSE | Mitochondrial pyruvate carrier 1 | AGALVRKAADYVR | -9.54 | 0.0187636 |
|  |  |  | GLPIAAINDMKKSPEIISGRMTFA | -25.48 | 0.000568434 |
| P63158 | HMGB1_MOUSE | High mobility group protein B1 | DPNAPKRPPSA | -8.52 | 0.004396917 |
| P68033 | ACTC_MOUSE | Actin, alpha cardiac muscle 1 | SKQEYDEAGPSIVH | -7.03 | 0.022258202 |
|  |  |  | TTGIVLDSGDGVTHNVPIYEGY | -12.12 | 0.010264602 |
| P68037 | UB2L3_MOUSE | Ubiquitin-conjugating enzyme E2 L3 | RNIQVDEANLLT | -6.36 | 0.000095153 |
| P70296 | PEBP1_MOUSE | Phosphatidylethanolamine-binding protein 1 | QAEWDDYVPKLYEQLSGK | -4.13 | 0.030307116 |
|  |  |  | RVDYAGVTVDELGKVLTPTQVM | -5.76 | 0.000726584 |
| P84089 | ERH_MOUSE | Enhancer of rudimentary homolog | SHTILLVQPTKR | -2.57 | 0.006961504 |
| P97450 | ATP5J_MOUSE | ATP synthase-coupling factor 6 | GEMDTFPTFK | -7.80 | 0.006483168 |
|  |  |  | KELDPVQK | 62.41 | 0.000532013 |
|  |  |  | PKFEVIDKPQS | 2.76 | 0.014183477 |
|  |  |  | RQASGGPVDIGPE | -7.82 | 0.000230653 |
| Q03265 | ATPA_MOUSE | ATP synthase subunit alpha | PVIETQAGDVSA | -21.45 | 0.010021675 |
| Q06185 | ATP5I_MOUSE | ATP synthase subunit e | VPPVQVSPLIKFGRYSALIIG | -110.10 | 0.011955298 |
| Q5EBG6 | HSPB6_MOUSE | Heat shock protein beta-6 | APSVALPTAQV | -12.41 | 0.002244946 |
|  |  |  | ASAPLPGFSAPGRLFDQ | -13.42 | 8.34629E-05 |
|  |  |  | PASAQAQLPSPPAAK | -3.32 | 0.005405737 |
|  |  |  | RAPSVALPTAQVSTDSGYFSVL | -12.44 | 0.022081037 |
|  |  |  | RAPSVALPTAQVSTDSGYFSVLL | -3.82 | 0.035843727 |
| Q60932 | VDAC1_MOUSE | Voltage-dependent anion-selective channel protein 1 | VNAGGHKLGLGLEFQA | -10.62 | 1.54974E-05 |
| Q60936 | COQ8A_MOUSE | Atypical kinase COQ8A | AVETHLQNLGLGGELL | -4.69 | 0.001606356 |
| Q61029 | LAP2B_MOUSE | Lamina-associated polypeptide 2, isoforms beta | SNEELLDQLVRY | -3.18 | 0.011638642 |
| Q61425 | HCDH_MOUSE | Hydroxyacyl-coenzyme A dehydrogenase | LDYVGLDTTKF | -8.16 | 0.001416789 |
| Q62167 | DDX3X_MOUSE | ATP-dependent RNA helicase DDX3X | SHVAVENALGLDQQ | -3.14 | 0.028261098 |
|  |  |  | SHVAVENALGLDQQF | -7.78 | 0.014281917 |
| Q62446 | FKBP3_MOUSE | Peptidyl-prolyl cis-trans isomerase FKBP3 | SEQVKNVKL | -15.58 | 0.028901651 |
| Q62448 | IF4G2_MOUSE | Eukaryotic translation initiation factor 4 gamma 2 | MESAIAEGGASRF | -2.95 | 0.036930792 |
| Q63918 | CAVN2_MOUSE | Caveolae-associated protein 2 | GEDAAQAEKF | -11.26 | 0.001566418 |
| Q64433 | CH10_MOUSE | 10 kDa heat shock protein | AAETVTKGGIMLPEKSQGKVLQA | -23.28 | 0.000376452 |
|  |  |  | AGQAFRKFLPLFDRVL | -51.60 | 0.00408621 |
|  |  |  | AGQAFRKFLPLFDRVLVER | 3.35 | 0.044860344 |
|  |  |  | FRDSDILGKYVD | -27.84 | 5.82385E-06 |
| Q6P8J7 | KCRS_MOUSE | Creatine kinase S-type | ASAFSKLLTGRNASL | 2.31 | 0.03462516 |
| Q8BFR5 | EFTU_MOUSE | Elongation factor Tu | TAAITKILAEGGGAK | -4.44 | 0.007058172 |
| Q8BK30 | NDUV3_MOUSE | NADH dehydrogenase flavoprotein 3 | DLNLDLSKF | -4.54 | 0.0001145 |
|  |  |  | LDLNLDLSK | -40.51 | 2.89479E-05 |
|  |  |  | LDLNLDLSKFRLPQPSSGRESPRH | -4.17 | 0.025527369 |
| Q8BWT1 | THIM_MOUSE | 3-ketoacyl-CoA thiolase, mitochondrial | AALSAGKVPPETIDSVIVGNVM | -17.54 | 0.005551234 |
|  |  |  | AARAALSAGKVPPETIDSVIVGNVM | -11.64 | 0.002041717 |
|  |  |  | AGKVPPETIDSVIVGNVM | -8.41 | 0.008307898 |
|  |  |  | KDFSATDLTEF | -5.99 | 6.74802E-06 |
|  |  |  | KDFSATDLTEFAARAA | -2.56 | 0.000818592 |
|  |  |  | RAALSAGKVPPETIDSVIVGNVM | -4.23 | 0.024294655 |
|  |  |  | RVGVPTETGALTLN | -3.80 | 0.040454921 |
|  |  |  | SATDLTEFAAR | -17.03 | 0.004640504 |
|  |  |  | SATDLTEFAARAALS | -18.70 | 0.004314039 |
|  |  |  | SGGAIALGHPLGGSGSR | -5.23 | 0.047515746 |
|  |  |  | VSGGAIALGHPLGGSGSRITA | -18.08 | 0.029860279 |
| Q8K1Z0 | COQ9_MOUSE | Ubiquinone biosynthesis protein COQ9 | STGEALVQGLMGAAVTLKNLTG | -6.20 | 0.01231762 |
| Q8K3J1 | NDUS8_MOUSE | NADH dehydrogenase iron-sulfur protein 8 | EAEIAANIQADYLYR | -2.79 | 0.004040251 |
|  |  |  | NIQADYLYR | -7.84 | 0.001212726 |
| Q8QZT1 | THIL_MOUSE | Acetyl-CoA acetyltransferase | GGVKLEDLIVKDGLTDV | -7.46 | 0.000788728 |
| Q8R3I3 | COG6_MOUSE | Conserved oligomeric Golgi complex subunit 6 | ADASGEVAAVPASGAAN | -2.84 | 0.005063595 |
| Q99JI1 | MSTN1_MOUSE | Musculoskeletal embryonic nuclear protein 1 | SEAGTPEGPIKK | -6.65 | 0.026386865 |
| Q99KI0 | ACON_MOUSE | Aconitate hydratase, mitochondrial | NAVTQEFGPVPDTAR | -7.10 | 0.046448086 |
| Q99LY9 | NDUS5_MOUSE | NADH dehydrogenase iron-sulfur protein 5 | PFLDIQKKLGISLDR | -7.48 | 0.03147592 |
| Q9CPQ3 | TOM22_MOUSE | Mitochondrial import receptor subunit TOM22 homolog | ILVLPVVFETEKLQ | -3.89 | 0.000311223 |
|  |  |  | RQILLGPNTGLSGGMPG | -3.79 | 0.001468506 |
| Q9CQ75 | NDUA2_MOUSE | NADH dehydrogenase 1 alpha subcomplex subunit 2 | AAAAASRAVGAKLG | -2.10 | 0.014392434 |
| Q9CQ91 | NDUA3_MOUSE | NADH dehydrogenase 1 alpha subcomplex subunit 3 | AGRISAFLKNAWAKEPVLVVSFS | -12.49 | 0.013684332 |
|  |  |  | TPYNYPVPVRDDG | -3.52 | 0.039841782 |
| Q9CQN3 | TOM6_MOUSE | Mitochondrial import receptor subunit TOM6 homolog | RNLSDIDLMAPQPGV | -4.55 | 2.07014E-05 |
| Q9CQQ7 | AT5F1_MOUSE | ATP synthase F(0) complex subunit B1 | PKTGVTGPYVLGTGLSLY | -5.87 | 0.033162721 |
|  |  |  | PLPPLPEYGGKVRLGLIPEEF | -13.77 | 0.028214 |
| Q9CQR2 | RS21_MOUSE | 40S ribosomal protein S21 | MQNDAGEFVDLYVPRK | -3.50 | 0.005865924 |
| Q9CQZ5 | NDUA6_MOUSE | NADH dehydrogenase 1 alpha subcomplex subunit 6 | AAAATGLRQAAA | -19.43 | 0.000627052 |
|  |  |  | AAAATGLRQAAAA | -13.86 | 0.000686559 |
|  |  |  | AAAATGLRQAAAAAASTSVKPI | -44.78 | 5.66769E-06 |
|  |  |  | AAAATGLRQAAAAAASTSVKPIFS | -7.32 | 1.79112E-05 |
| Q9CR21 | ACPM_MOUSE | Acyl carrier protein, mitochondrial | SDAPPLTLDGIKDRVLYV | -5.94 | 0.007522356 |
|  |  |  | AARSGPFAPVLSATSRGVAGALR | 2.53 | 0.001516736 |
|  |  |  | ARPLVATVGLNVPASVR | -34.92 | 0.000125078 |
|  |  |  | GQAAARPLVATV | -9.79 | 0.000155305 |
|  |  |  | GVAGALRPLLQGAVPAASEPPVLDV | -12.83 | 0.025648975 |
|  |  |  | LSGQAAARPLVATVGLNVPASVR | -3.70 | 0.002472143 |
|  |  |  | MLSVAARSG | 5.97 | 0.00976142 |
|  |  |  | MLSVAARSGPFAPVLSA | 2.11 | 0.001931207 |
|  |  |  | MLSVAARSGPFAPVLSATSRGVA | -5.24 | 0.007555803 |
|  |  |  | PLVATVGLNVPAS | -4.54 | 5.26406E-05 |
|  |  |  | VATVGLNVPASVR | -6.94 | 0.009996327 |
| Q9CRB6 | TPPP3_MOUSE | Tubulin polymerization-promoting protein family member 3 | AASTDIAGLEESFRKF | -7.78 | 0.004308494 |
| Q9CXZ1 | NDUS4_MOUSE | NADH dehydrogenase iron-sulfur protein 4 | ADNQTRDTQLITV | -3.13 | 0.047388467 |
|  |  |  | ASTADPLSNMVLTF | -45.08 | 0.00100741 |
|  |  |  | ITVDEKLDITTL | -19.06 | 0.025830081 |
| Q9CZU6 | CISY_MOUSE | Citrate synthase, mitochondrial | AAAMNGLAGPLHGLANQEV | -7.37 | 0.011051663 |
|  |  |  | LVYETSVLDPDEGIR | -3.55 | 0.011901927 |
|  |  |  | NSESNFARAY | -11.10 | 4.33171E-06 |
|  |  |  | NSGRVVPGYGHAVLR | -5.29 | 0.00372515 |
|  |  |  | SFAAAMNGLAGPLHGLANQEV | -3.87 | 0.000642376 |
| Q9D2G2 | ODO2_MOUSE | 2-oxoglutarate dehydrogenase complex component E2 | IDISVAVATPRGLVVPV | -8.42 | 0.002172265 |
| Q9D3D9 | ATPD_MOUSE | ATP synthase subunit delta, mitochondrial | SPTQVFFDSA | 2.80 | 0.030341086 |
| Q9D6J6 | NDUV2_MOUSE | NADH dehydrogenase flavoprotein 2 | AEVLQVPPMRVYEV | -5.06 | 0.001957367 |
| Q9D855 | QCR7_MOUSE | Cytochrome b-c1 complex subunit 7 | AGRSAVSASSKWLDGFRKWYY | 3.14 | 0.008984766 |
| Q9DBG5 | PLIN3_MOUSE | Perilipin-3 | VGPFAPGITEKTPEGK | -16.03 | 0.028727454 |
| Q9DC77 | SMPX_MOUSE | Small muscular protein | SKQPISNVR | -97.66 | 0.000262933 |
|  |  |  | SKQPISNVRA | -8.91 | 0.008345395 |
|  |  |  | SKQPISNVRAI | -10.28 | 0.035388512 |
| Q9DCT8 | CRIP2_MOUSE | Cysteine-rich protein 2 | IYEKPQTEAPQVTGPIEVPVVR | -12.14 | 0.001118382 |
|  |  |  | PKGVNTGAVGSYIYDKDPEGTVQP | -11.29 | 0.008234599 |
|  |  |  | VGSYIYDKDPEGTVQP | -3.98 | 0.019744092 |
| Q9DCX2 | ATP5H_MOUSE | ATP synthase subunit d | ALKTIDWVSF | -3.56 | 0.029593082 |
|  |  |  | ASLSEKPPAIDWA | 4.06 | 0.046347806 |
|  |  |  | ASLSEKPPAIDWAYY | -15.85 | 0.000456622 |
|  |  |  | KSWNETFHARLASL | 2.75 | 0.042839805 |
|  |  |  | NALKIPVPEDK | -10.19 | 0.032048689 |
|  |  |  | SWNETFHARLASL | -7.95 | 0.001137858 |
|  |  |  | VEVMPQNQK | -19.37 | 0.006612023 |
|  |  |  | VEVMPQNQKAIGN | -21.23 | 8.36801E-05 |
|  |  |  | VEVMPQNQKAIGNAL | -3.59 | 0.044328291 |
|  |  |  | VEVMPQNQKAIGNALKSWNETF | -19.14 | 0.005931605 |
| Q9JKS4 | LDB3_MOUSE | LIM domain-binding protein 3 | KDLAVDSASPV | -11.94 | 0.000565983 |
|  |  |  | PAYNPTGPQVTPLAR | -14.63 | 0.007998833 |
|  |  |  | PRGAPAYNPTGPQVTPLAR | -4.95 | 0.036583803 |
|  |  |  | PRGAPAYNPTGPQVTPLARGTFQRA | -12.84 | 0.003307274 |
|  |  |  | SGPVLPLGSPVAK | -7.08 | 0.002749477 |
| Q9JLV1 | BAG3_MOUSE | BAG family molecular chaperone regulator 3 | SAATQSPMMQMA | -37.62 | 0.009493648 |
| Q9QXV0 | PCS1N_MOUSE | ProSAAS (IA-4) | SLSAASAPLVETSTPLRL | -5.73 | 0.000728363 |
| Q9R0Y5 | KAD1_MOUSE | Adenylate kinase isoenzyme 1 | GYTHLSTGDLL | -16.18 | 0.000898278 |
|  |  |  | NATEPVISF | -10.90 | 0.011665683 |
| Q9WVA2 | TIM8A_MOUSE | Mitochondrial import inner membrane translocase subunit Tim8 A | MESSTSSSGSALGAVDPQLQHF | -8.56 | 0.040306654 |
| Q9WVC3 | CAV2_MOUSE | Caveolin-2 | GLETEKADVQLF | -8.67 | 0.001237661 |
| Q9Z1P6 | NDUA7_MOUSE | NADH dehydrogenase 1 alpha subcomplex subunit 7 | ASATRVIQKLRNW | 2.07 | 0.024068913 |
|  |  |  | ASGQDLQAKLQ | -15.49 | 0.001150183 |

**Supple. Table 2.  Identification of unique peptides**

| **Peptide Sequence** | **Uniprot Accession** | **Protein** | **Fold Change** | **p Value** |
| --- | --- | --- | --- | --- |
| **Unique peptides in control group** | | | | |
| SNPPLNGARIAATIL | P05202 | AATM_MOUSE | -13.42243864 | 0.0011950700 |
| KVDKGVVPL | P05064 | ALDOA_MOUSE | -20.29299349 | 0.0091542710 |
| VEVMPQNQKAIGN | Q9DCX2 | ATP5H_MOUSE | -21.22577447 | 0.0000836801 |
| VEVMPQNQKAIGNALKSWNETF | Q9DCX2 | ATP5H_MOUSE | -19.13587736 | 0.0059316050 |
| PVIETQAGDVSA | Q03265 | ATPA_MOUSE | -21.44962853 | 0.0100216750 |
| SAATQSPMMQMA | Q9JLV1 | BAG3_MOUSE | -37.62430506 | 0.0094936480 |
| AAETVTKGGIMLPEKSQGKVLQA | Q64433 | CH10_MOUSE | -23.28237373 | 0.0003764520 |
| AGQAFRKFLPLFDRVL | Q64433 | CH10_MOUSE | -51.59914523 | 0.0040862100 |
| FRDSDILGKYVD | Q64433 | CH10_MOUSE | -27.8386307 | 0.0000058239 |
| EMVPLKYFENWSASMI | P00405 | COX2_MOUSE | -40.55318272 | 0.0315895250 |
| FGEHLLESDLF | P23927 | CRYAB_MOUSE | -24.10368983 | 0.0012712440 |
| SPFYLRPPSFLR | P23927 | CRYAB_MOUSE | -9.657465163 | 0.0032786270 |
| SPFYLRPPSFLRAPS | P23927 | CRYAB_MOUSE | -9.100662188 | 0.0003158850 |
| KPFAWGDGNHTL | P43023 | CX6A2_MOUSE | -12.76846104 | 0.0063505810 |
| SAAKGDHGGAGANTWRLLTFVL | P43023 | CX6A2_MOUSE | -47.58651883 | 0.0008128340 |
| LENPKKYIPGTKMIF | P62897 | CYC_MOUSE | -20.23628289 | 0.0077961370 |
| MEYLENPKKYIPGTK | P62897 | CYC_MOUSE | -27.70923667 | 0.0024080310 |
| TDANKNKGITWGEDT | P62897 | CYC_MOUSE | -48.95706999 | 0.0176110420 |
| TDANKNKGITWGEDTLME | P62897 | CYC_MOUSE | -171.2512334 | 0.0148282940 |
| GFGDLKTPAGLQVLNDYL | O70251 | EF1B_MOUSE | -15.39016745 | 0.0016000720 |
| ADAFVGTWKLVDSKNF | P11404 | FABPH_MOUSE | -8.135949106 | 0.0006199220 |
| VDSKNFDDYMKSLGVGF | P11404 | FABPH_MOUSE | -12.79538433 | 0.0095745160 |
| AWGKIGGHGAEYGAEAL | P01942 | HBA_MOUSE | -24.27003913 | 0.0202049600 |
| SESGSKSSQPL | P17095 | HMGA1_MOUSE | -19.99896445 | 0.0026297080 |
| DPNAPKRPPSA | P63158 | HMGB1_MOUSE | -8.515603946 | 0.0043969170 |
| AEGPAAVTLAAPAFSRALNRQL | P14602 | HSPB1_MOUSE | -7.884835729 | 0.0073110240 |
| GWPGYVRPLPAATAEGPAAVTL | P14602 | HSPB1_MOUSE | -28.51585118 | 0.0022733030 |
| ASAPLPGFSAPGRLFDQ | Q5EBG6 | HSPB6_MOUSE | -13.41898997 | 0.0000834629 |
| TTPTVSKATSPSTLVSTGPSSR | P27546 | MAP4_MOUSE | -20.00326573 | 0.0040569810 |
| AKVAVLGASGGIGQPLSLL | P08249 | MDHM_MOUSE | -14.38495941 | 0.0016243390 |
| GRVNVKNEEIDEMIKEAPGPINF | P51667 | MLRV_MOUSE | -22.71076407 | 0.0009687690 |
| KEAPGPINFTVF | P51667 | MLRV_MOUSE | -14.90375557 | 0.0010387840 |
| LTMFGEKLKGADPEETILNA | P51667 | MLRV_MOUSE | -35.90689672 | 0.0000176504 |
| VKNEEIDEMIKEAPGPINF | P51667 | MLRV_MOUSE | -23.5820975 | 0.0191314950 |
| VKNEEIDEMIKEAPGPINFT | P51667 | MLRV_MOUSE | -34.9507235 | 0.0000083462 |
| GLPIAAINDMKKSPEIISGRMTFA | P63030 | MPC1_MOUSE | -25.48307111 | 0.0005684340 |
| LLFDRTPKGEMKITY | P09542 | MYL3_MOUSE | -35.80356702 | 0.0266827080 |
| AAAATGLRQAAAAAASTSVKPI | Q9CQZ5 | NDUA6_MOUSE | -44.77583137 | 0.0000056677 |
| ASTADPLSNMVLTF | Q9CXZ1 | NDUS4_MOUSE | -45.07657225 | 0.0010074100 |
| ITVDEKLDITTL | Q9CXZ1 | NDUS4_MOUSE | -19.05798369 | 0.0258300810 |
| LDLNLDLSK | Q8BK30 | NDUV3_MOUSE | -40.50970444 | 0.0000289479 |
| LTTSPKLITGLTM | P03911 | NU4M_MOUSE | -51.92614047 | 0.0048309350 |
| FDITADDEPLGRVSFEL | P17742 | PPIA_MOUSE | -33.66636664 | 0.0052393180 |
| SKQPISNVR | Q9DC77 | SMPX_MOUSE | -97.6604845 | 0.0002629330 |
| QQKYEINVL | P50752 | TNNT2_MOUSE | -93.46418943 | 0.0113876800 |
| APPTAYIDFA | P17751 | TPIS_MOUSE | -16.49112252 | 0.0009379440 |
| ARPLVATVGLNVPASVR | Q9CR68 | UCRI_MOUSE | -34.91811527 | 0.0001250780 |
| **Unique peptides in DOX group** | | | | |
| APAFSRAL | P14602 | HSPB1_MOUSE | 293.2250219 | 0.0000003644 |
| AEGPAAVTLAAPAFSRAL | P14602 | HSPB1_MOUSE | 4.970079013 | 0.0279771700 |
| KELDPVQK | P97450 | ATP5J_MOUSE | 62.41098066 | 0.0005320130 |
| QFGLHPWLPSAM | P03921 | NU5M_MOUSE | 4.550171438 | 0.0099827830 |
| RLLTFVLALPGVA | P43023 | CX6A2_MOUSE | 67.6514452 | 0.0008900470 |

**Supple.Table 3. The same peptides derived from several precursor proteins**

| **Peptide Sequence** | **Protein** | **Fold Change** | **p Value** |
| --- | --- | --- | --- |
| VEVMPQNQK | Atp5pd | -19.3683617 | 0.006612023 |
|  | mCG6620 |  |  |
|  | mCG55033 |  |  |
| GEHTPSALAI | Aldo1 | -14.86572408 | 0.000533499 |
|  | mCG22383 |  |  |
| GEMDTFPTFK | Atp5pf | -7.801903978 | 0.006483168 |
|  | mCG132409 |  |  |
